# Constitutive Function of the Ikaros Transcription Factor in Primary Leukemia Cells from Pediatric Newly Diagnosed High-Risk and Relapsed B-precursor ALL Patients

**DOI:** 10.1371/journal.pone.0080732

**Published:** 2013-11-20

**Authors:** Fatih M. Uckun, Hong Ma, Rita Ishkhanian, Martha Arellano, Anoush Shahidzadeh, Amanda Termuhlen, Paul S. Gaynon, Sanjive Qazi

**Affiliations:** 1 Systems Immunobiology Laboratory and Developmental Therapeutics Program, Children’s Center for Cancer and Blood Diseases, Children’s Hospital Los Angeles, Los Angeles, California, United States of America; 2 Department of Pediatrics, University of Southern California Keck School of Medicine, Los Angeles, California, United States of America; 3 Developmental Therapeutics Program, USC Norris Comprehensive Cancer Center, Los Angeles, California, United States of America; 4 Jonathan Jaques Cancer Center, Miller Children’s Hospital, Long Beach, California, United States of America; Josep Carreras Leukaemia Research Institute, University of Barcelona, Spain

## Abstract

We examined the constitutive function of the Ikaros (IK) transcription factor in blast cells from pediatric B-precursor acute lymphoblastic leukemia (BPL) patients using multiple assay platforms and bioinformatics tools. We found no evidence of diminished IK expression or function for primary cells from high-risk BPL patients including a Philadelphia chromosome (Ph)^+^ subset. Relapse clones as well as very aggressive *in vivo* clonogenic leukemic B-cell precursors isolated from spleens of xenografted NOD/SCID mice that developed overt leukemia after inoculation with primary leukemic cells of patients with BPL invariably and abundantly expressed intact IK protein. These results demonstrate that a lost or diminished IK function is not a characteristic feature of leukemic cells in Ph^+^ or Ph^-^ high-risk BPL.

## Introduction

Ikaros (IK) is a zinc finger (ZF)-containing sequence-specific DNA-binding protein encoded by the *IKZF1* gene. It plays a pivotal role in immune homeostasis through transcriptional regulation of the earliest stages of lymphocyte ontogeny and differentiation by both (a) gene transcriptional activation via efficient transcription initiation and elongation as well as (b) repression [1]. In recent years, single-nucleotide polymorphism (SNP) arrays have been used to evaluate the incidence and prognostic significance of *IKZF1* deletions in primary leukemic cells from pediatric patients with high-risk B-precursor ALL (BPL) [2-7]. While discrepancies with respect to the incidence of genomic *IKZF1* deletions as well as their independent prognostic impact have been noted [3-7], it has generally been assumed that *IKZF1* is a haploinsufficient gene rendering its hemizygous deletions in ALL cells biologically and clinically significant by causing IK deficiency at the cellular level [2-7]. However, the reported SNP array based *IKZF1* deletion data were not accompanied by functional data on expression levels of IK target genes or EMSA of IK-specific DNA binding activity in nuclear extracts to support the functional IK deficiency proposal [3,4]. A more recent report by Palmi et al. suggested that the prognostic significance of *IKZF1* deletions is markedly enhanced when additional copy number abnormalities involving other genes are present [7], which prompts the hypothesis that the observed association of *IKZF1* deletions with poor treatment outcome may stem from a profound underlying genomic instability rather than lost or diminished IK function.

The purpose of the present study was to examine the expression and function of IK protein in leukemia cells from high-risk pediatric BPL patients using multiple assay platforms and bioinformatics tools. Our study was designed to determine if BPL cells are deficient for IK function as originally proposed [2-4]. In order to evaluate IK function in BPL cells, we examined in patient-derived leukemia cells (i) transcript expression levels of IK target genes and IK-regulated lymphoid priming genes, (ii) protein expression levels of IK in whole cell lysates and nuclear protein extracts, (iii) subcellular localization of IK (nuclear localization requires DNA binding function of IK), and (iv) DNA binding activity of native IK. We found no evidence of diminished IK expression or function in primary cells from high-risk BPL patients, including a Ph^+^ subset, with any of the assay platforms used. To the contrary, IK function appeared to be highest in the Ph^+^ subset. To our surprise, relapse clones as well as aggressive xenograft ALL cells also expressed abundant levels of IK.

## Materials and Methods

### Patient Cells and Animal Research

Cryopreserved primary leukemia cells from pediatric BPL patients as well as BPL xenograft cells isolated from spleen specimens of xenografted NOD/SCID mice were used in the described experiments (File **S1**). The IRB (CCI) at Children’s Hospital Los Angeles (CHLA) (Human Subject Assurance Number: FWA0001914) determined that the use of leukemic cells in our research did not meet the definition of human subject research per 45 CFR 46.102 (d and f) since it does not include identifiable private information. The research was approved by the CHLA IRB/CCI. The IRB approved project numbers were CCI-09-00304 (CCI Review Date 12/21/2009, Approval Date: 12/29/09) for cryopreserved cells and CCI-10-00141 (CCI Review Date 7/27/2010, Approval Date 7/27/2010) for freshly obtained primary leukemia cells. We also used an NOD/SCID mouse model of human B-precursor ALL [2]. NOD/SCID mice (NOD.CB17-*Prkdc^scid^*/J; 4-6 weeks of age at the time of purchase, female) were obtained from the Jackson Laboratory (Sacramento, CA). The research was conducted according to Institutional Animal Care and Use Committee (IACUC) Protocol 280-09, that was approved by the IACUC of CHLA on 11-24-2009 and its 3-year rewrite application 280-12 that was approved on 7-10-2012. All animal care procedures conformed to the Guide for the Care and Use of Laboratory Animals (National Research Council, National Academy Press, Washington DC 1996, USA).

### Bioinformatics and Statistical Analysis of Gene Expression Profiles

The publically available archived GSE32311 database [8] was used to compare gene expression changes in control thymocytes from *IKZF1* wildtype mice (GSM800500, GSM800501, GSM800502) vs. IK-deficient thymocytes from *IKZF1* null mice (GSM800503, GSM800504, GSM800505) with the same genetic background of (C57BL/6 x129S4/SvJae). The Gene Pattern software (http://www.broadinstitute.org/cancer/software/genepattern) was utilized to extract expression values for human lymphocyte precursors from the National Center for Biotechnology Information (NCBI) Gene Expression Omnibus (GEO) database [9]. We compiled the archived gene expression profiling (GEP) data on primary leukemia specimens from 1486 pediatric acute lymphoblastic leukemia (ALL) patients across 7 independent studies (GSE3912, N=113; GSE18497, N=82; GSE4698, N=60; GSE7440, N=99; GSE13159, N=750; GSE11877, N=207; GSE12995, N=175) that combined datasets from U133A and U133 Plus 2 genechips using common *IKZF1* probe sets to focus our analysis on 45 validated IK target genes as well as 20 IK-regulated lymphoid-priming genes [10] (File **S1**; Table **S1** and Table **S2** in File **S1**). In addition, we compiled 6 archived gene expression profiling datasets that measured expression from B-precursor ALL childhood patients hybridized to Human Genome U133 Plus 2.0 Array containing the 10 *IKZF1* probe sets for determination of exon specific expression (GSE11877, N=207; GSE13159 N=823; GSE13351 N=107; GSE18497, N=82, GSE28460, N=98; GSE7440, N=99; Total, N=1416) (File **S1**). Further, we performed gene set enrichment analyses (GSEA) in which rank ordered differences in standard deviation units for BCR-ABL^+^ BPL samples (N=20) compared to other samples (N=155) in the Mullighan study (GSE12995) and Ph^+^ BPL (N=122) versus Ph- BPL (N=237) in the “The Microarray Innovations in Leukemia” (MILE) study (GSE13159) were processed for enrichment of lymphoid priming genes (18 genes represented on the gene chips) and IK target genes (39 genes represented in the Mullighan study, 45 genes represented in the MILE study) using a supervised approach implemented in GSEA v2.08 (Broad institute) (File **S1**). These ranked ordered genes were screened for enrichment of gene sets in 13321 genes (22283 transcripts) for the Mullighan study and 20606 genes (54613 transcripts) for the MILE study using weighted Kolmogorov-Smirnov statistics implemented in GSEA (GSEA v2.08 (Broad Institute).

### Standard Biochemical Assays, PCR, Confocal Imaging, and Transfection Methods

Confocal Laser Scanning Microscopy, Western blot analyses, and electrophoretic mobility shift assays (EMSA) were performed as per previously described standard procedures (Data Supplement). We also performed PCR and real-time quantitative PCR on genomic DNA samples using standard procedures (File **S1**). The primer sets used for amplifying and sequencing *Ikaros/IKZF1* exons 4, 5, 6 and 7 and their exon-intron junctions are listed in Table **S3** in File **S1**.

### SCID Mouse Xenograft Model of Human BPL

We used an NOD/SCID mouse model of human BPL to study IK expression levels in *in vivo* clonogenic ALL xenograft cells. The derivation and immunophenotypic features of the xenograft cells were recently published [11].

## Results

### Examination of Primary Leukemia Cells from Ph^+^ High-Risk BPL Patients for Structural and Functional Integrity of *IKZF1* Gene using Genomic PCR, RT-PCR, Western Blot Analysis, and EMSA

**Figure 1:**
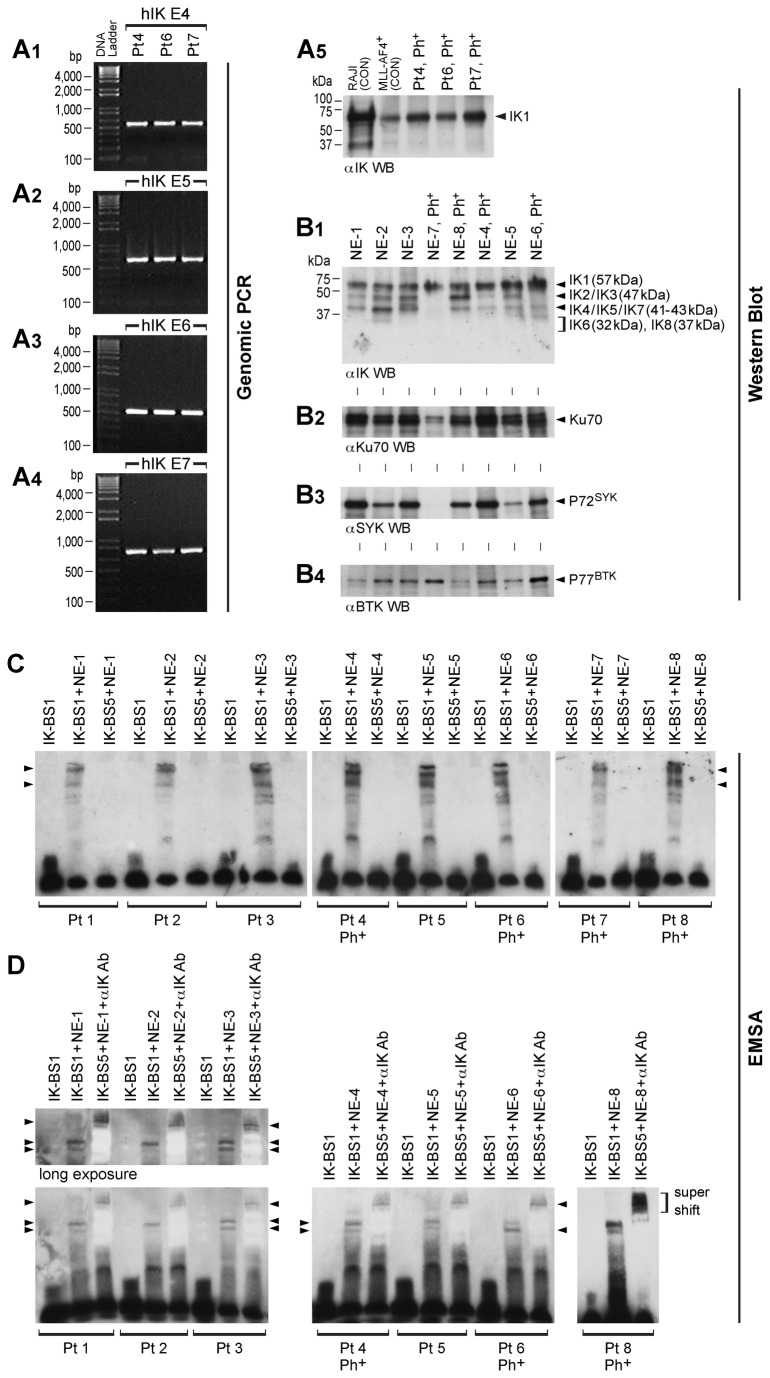
Pediatric Ph^+^ BPL is not Characterized by IK-deficiency. **[A1-A4] Genomic PCR**. **Analysis of the Human *Ikaros*/*IKFZ1* Gene Exons E4-E7 in High-Risk BPL**. We performed exon-specific IKZF1 PCR with DNA sequencing on purified genomic DNA samples from 3 pediatric patients with Ph^+^ high-risk BPL. Exons E4-E7 and their intron-exon junctions were PCR amplified using the PCR primers in Table **S3** in File **S1**. Depicted are agarose gels documenting that normal size PCR products (E4: 577-bp, E5: 646-bp, E6: 530-bp, E7:785-bp) for each of the 4 IKZF1 exons were obtained in each of the 3 patients, providing strong evidence against the existence of homozygous deletions of the entire IKZF1 locus or within. IKZF1 exons E4-E7. **[A5] Western Blot Analysis of Ikaros Expression in Ph^+^ High-Risk**. **BPL**. Depicted is an anti-IK Western blot of whole cell lysates of primary leukemia cells from 3. Ph^+^ BPL patients. Similar to RAJI cells and primary cells from an MLL-AF4^+^ ALL case that were included as controls, Ph^+^ ALL cells from each of the 3 cases expressed an intact 57 kDa IK1 protein. **[B] Nuclear Expression of Intact Ikaros Protein and Its Regulators in Ph^+^ and Ph^-^ High-Risk BPL**. Depicted are Western blots of nuclear protein extracts (NE) from primary leukemic cells of 8 high-risk pediatric BPL patients, including 4 Ph^+^ ALL cases. NE were examined for presence of IK (B1), Ku70 (B2), SYK (B3) and BTK (B4). See text for discussion. **[C & D] Assessment of Sequence-Specific DNA Binding Function of Nuclear Ikaros in Ph^+^ and Ph^-^ High-Risk BPL**. Electrophorectic mobility shift assays (EMSA) were performed on nuclear extracts from BPL cells using the Thermo Scientific LightShift Chemiluminescent EMSA Kit and the *IK-BS1* test probe containing a high-affinity IK1 binding site and the *IK*-*BS5* negative control probe that has a single base pair (G>A) substitution at position 3 within the core consensus and does not bind IK [10], [12], [13]. The nuclear protein extracts from each of the 4 Ph^+^ and 4 Ph^-^ BPL cases showed significant gel retardation of the IK1-specific IK-BS1 probe (but not the control IK-BS5 probe) (**C**). Supershift assays were performed in 3 Ph^+^ and 4 Ph^-^ BPL cases with an anti-IK monoclonal antibody (2 µg/sample) to confirm the presence of IK in the retarded DNA-binding protein complexes, as previously described [12] (**D**). Positions of the retarded and supershifted bands are indicated with arrowheads. In each case, the retarded complex of the IK-BS1 probe was supershifted with the anti-IK antibody.

In an effort to identify homozygous *IKZF1* deletions, we performed exon-specific genomic PCR with DNA sequencing on purified genomic DNA samples from 3 pediatric patients with Ph^+^ high risk BPL. As shown in Figure **1A1-A4**, no homozygous deletions involving Exon 4, Exon 5, Exon 6 or Exon-7 were found in any of the 3 cases. This absence of genomic deletions in the E4-E7 segment encoding the DNA binding domain of IK prompted us to further validate our findings using Western blot analyses with an IK-specific antibody. Leukemic cells from all 3 Ph^+^ BPL cases expressed a ~57-kDa immunoreactive protein corresponding in size to full-length IK1 (Figure **1A5**). We next sought to determine if the detected IK1 protein in Ph^+^ BPL cells enters the nucleus and binds to DNA in a sequence-specific manner. As shown in Figure **1B**, Ph^+^ BPL cells from each of the 4 cases (3 cases from Panel A plus an additional Ph^+^ BPL case) examined showed nuclear expression of DNA binding IK isoform IK1 (57 kDa) as well as at least 2 of the 3 IK-activating regulatory proteins SYK [10], Ku70 [12] and BTK [13]. Likewise, nuclear expression of IK and IK-activating regulatory proteins was confirmed for 4 of 4 Ph^-^ ALL cases (Figure **1B**). In EMSA tests, the nuclear protein extracts of Ph^+^ BPL cells caused selective gel retardation of the *IK-BS1* test probe containing a high-affinity IK1 binding site (Figure **1C**). The presence of native IK in these in retarded DNA-binding protein complexes was confirmed in supershift assays using an IK-specific monoclonal antibody (Figure **1D**). These findings provided compelling evidence that primary leukemia cells from Ph^+^ (as well as Ph^-^) pediatric high-risk BPL patients express a fully functional IK protein, thereby confirming and extending the results of the genomic PCR and Western blot assays.

### Examination of Leukemia Cells from Patients with Newly Diagnosed Pediatric High Risk BPL, Including Its Ph^+^ Subset, for *IKZF1* Deletions by Real-time Quantitative PCR Analysis of the *Ikaros/IKZF1* Gene

**Figure 2:**
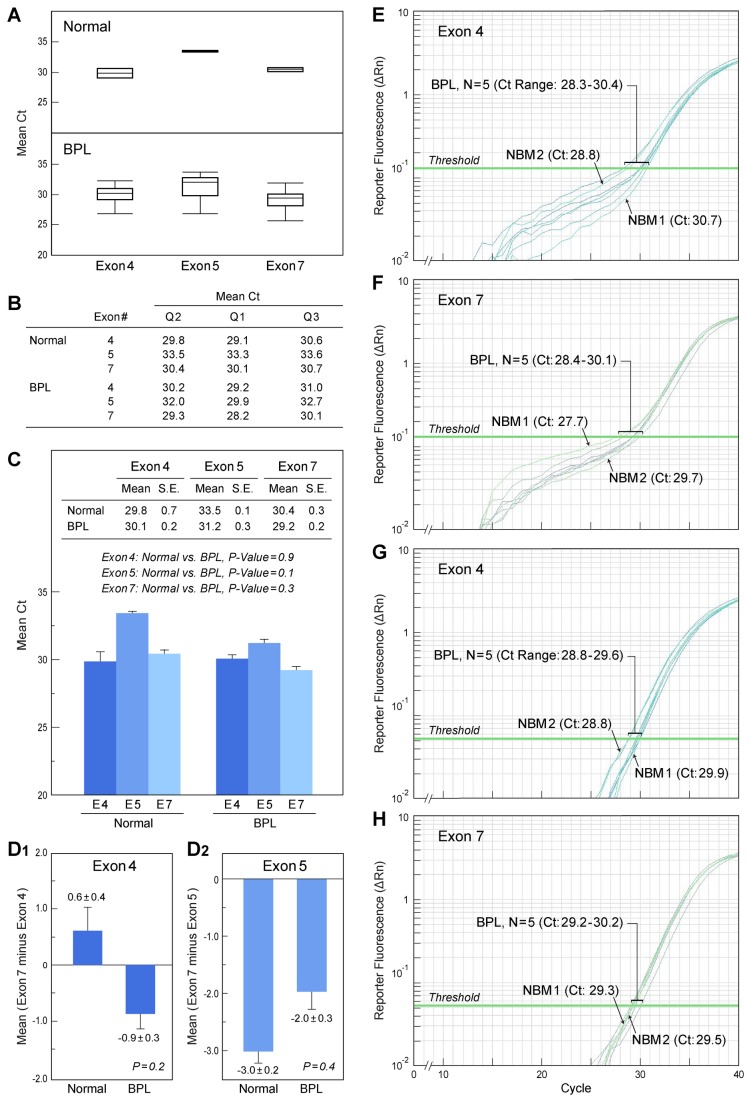
Evaluation of Primary Leukemic Cells from High-Risk Pediatric BPL Patients for IKZF1 Deletions Involving Exons 4 or 5 Using Real-time Quantitative PCR. **[A]** Depicted are the Box- and Whisker plots of the mean Ct values from 2 independent technical replicates of exon-specific IKZF1 qPCR performed on genomic DNA of primary leukemic cells from 32 BPL patients and 2 normal control bone marrow specimens. In each PCR reaction, the input of the template was standardized by using 105 ng of genomic DNA. In addition, a reference primer pair for Exon 7 was included for normalization of the qPCR data for Exon 4 and Exon 5. There were no outliers for Ct values for IKZF1 Exons 4, 5 or 7. No BPL case was identified by interpatient comparisons or comparisons between leukemic and normal samples that had a significantly higher Ct value suggestive of an IKZF1 deletion. **[B]** The Q2 (Median), Q1 (25^th^ Percentile/Lower quartile) and Q3 (75^th^ Percentile/Upper quartile) values for the box plots shown in [A]. **[C]** Bar graphs of Ct data from exon-specific IKZF1 qPCR for 32 BPL patients averaged across 2 independent experiments and then compared with Ct data for normal hematopoietic cells from 2 normal bone marrow specimens averaged from 3 independent experiments in which they were included as controls for side-by-side comparison with leukemic specimens from BPL patients (2 experiments) or BPL xenograft cells (one experiment). The inset shows the mean±SE values. No statistically significant differences were observed between normal vs. BPL specimens as documented by the P-values shown. **[D]** Bar graphs of Ct data from [C] for Exon 4-specific qPCR and Exon 5-specific qPCR after normalization by referencing the raw data to the Ct values from Exon 7-specific qPCR. The data normalization employed the method reported by Weksberg et al [15] and Moody et al [16] (File **S1**). **[E-H]** Depicted are representative cycle number vs. Log (∆Rn) (fluorescence signal) amplification plots obtained using the IKZF1 Exon 4-specific test primers and IKZF1 Exon 7-specific reference primers for the normal hematopoietic cells and randomly picked 5 BPL cases.

It has been reported that the most common *IKZF1* microdeletions in Ph^+^ BPL occur between Exons 4-7 (30%) or Exons 2-7 (15%) [14]. In particular, deletions of Exons 4-7 has been proposed as the molecular cause for the generation of IK6, a non-DNA binding truncated IK isoform that lacks all of the DNA binding domains of IK1. Exons 4 and 5 contain 3 of the 4 zinc fingers and their absence is a characteristic feature of the non-DNA binding IK isoforms IK4, IK5, IK6, IK7, IK8, and IK9. We therefore used real-time qPCR to examine side-by-side genomic DNA from normal hematopoietic cells in normal bone marrow specimens (N=2) and primary leukemic cells from 32 pediatric patients with high-risk BPL, including 4 Ph^+^ ALL patients, for evidence of intragenic deletions of *IKZF1* involving Exon 4 or Exon 5. *IKZF1* Exon 7 was also amplified as an internal reference control. No Ph^+^ or Ph^-^ high-risk BPL case was identified by interpatient comparisons to have a significantly higher “outlier” Ct value suggestive of an *IKZF1* deletion (Figure **2** **, Panels A-C**). Likewise, none of the leukemic samples had a significantly higher raw (Figure **2** **, Panels A-C**) or normalized (Figure **2** **, Panels D1 & D2**) Ct value higher than the median Ct value of the normal samples. Representative *IKZF1* Exon 4-specific (and Exon 7-specific) qPCR amplification plots are depicted in Figure **2** **, Panels E-H**. These results demonstrate that *IKZF1* deletions are not consistent abnormalities in pediatric high-risk BPL and they are not signature characteristics of pediatric Ph^+^ BPL, as previously reported [3].

### Examination of Leukemia Cells from Patients with Newly Diagnosed Pediatric High Risk BPL, Including Its Ph^+^ Subset, for *IKZF1* Deletions by Gene Expression Analysis Using Multiple *IKZF1* Probesets

**Figure 3:**
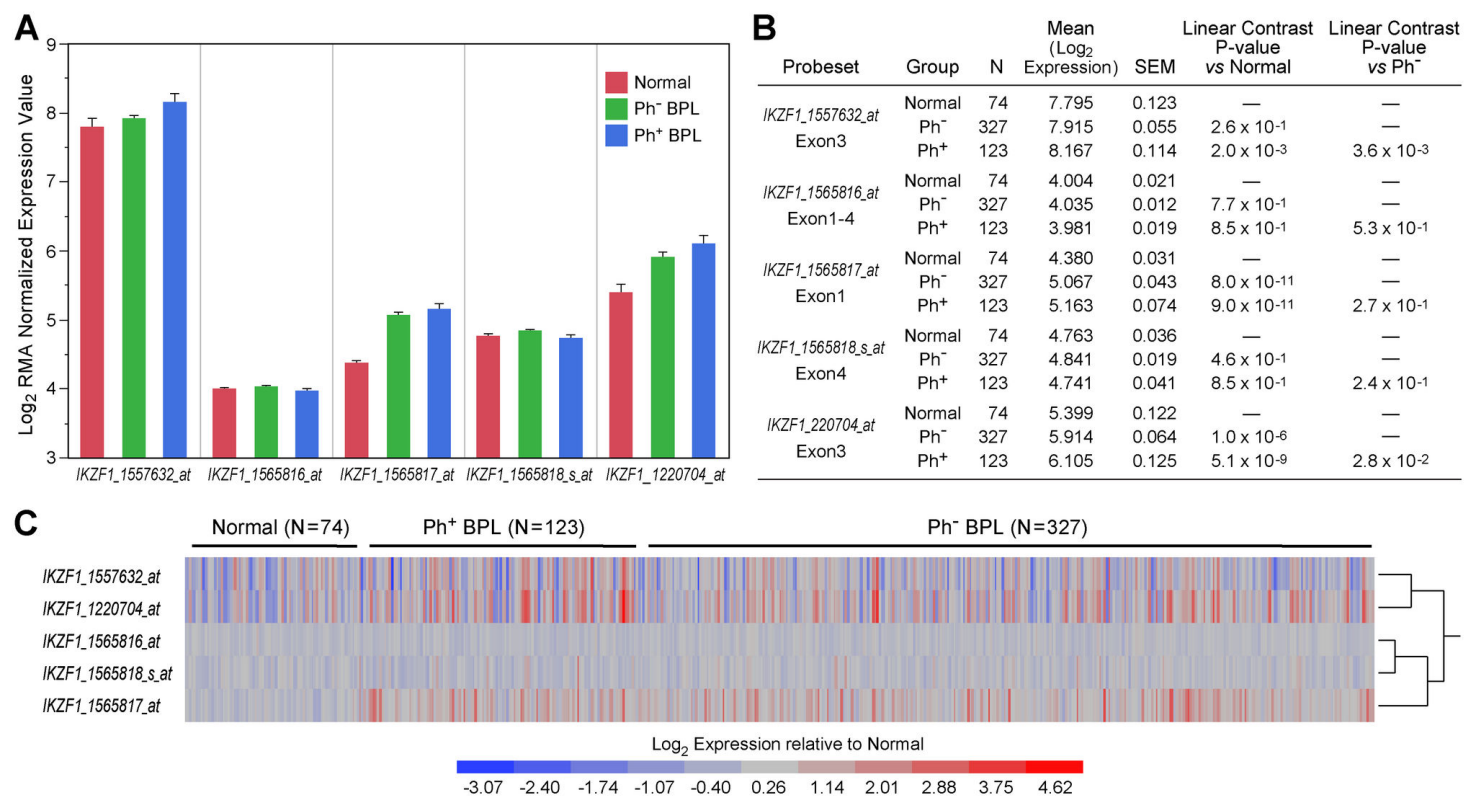
Exon-specific Detection of IKZF1 Transcripts in Normal Hematopoietic Cells and Primary Leukemic Cells from Pediatric Ph^+^ vs. Ph^-^ BPL Patients. IKZF1 transcript levels, as measured by 5 IKZF1 probe sets specific for IKZF1 Exons 1-4, were compared between primary leukemic cells from 123 pediatric Ph^+^ BPL patients, 327 pediatric Ph^-^ BPL patients and non-leukemic hematopoietic cells from 74 normal bone marrow specimens analyzed in 2 independent studies (viz.: GSE13159 and GSE13351). Depicted in (**A**) are bar charts of RMA-normalized transcript levels for the 5 IKZF1 probe sets, including 2 probe sets exhibiting Exon 4 specificity to test for reduction in signal due to an intragenic IKZF1 deletion involving a region within Exons 4-7 or Exons 2-7. No significant reduction in expression levels were observed for these 2 Exon 4 probesets (**B**) and significant increases in expression were observed for both comparisons of Ph^+^ with Normal and Ph^-^ samples for the two Exon 3 probesets. Heat map depicts up- and down-regulated transcripts ranging from red to green respectively for RMA-normalized IKZF1 transcript levels of leukemic cells mean centered to normal hematopoietic cells and clustered according to the average distance metric (**C**).

In order to gain further insights into the incidence and biological significance of *IKZF1* deletions, we examined the expression levels of *IKZF1* transcripts in primary leukemic cells from 327 pediatric Ph^-^ BPL patients in side by side comparison with 123 pediatric Ph^+^ BPL cases and 74 normal bone marrow specimens obtained from the National Center for Biotechnology Information (NCBI) Gene Expression Omnibus (GEO) database GSE13159 and GSE13351. BLAT analysis on *IKZF1* target sequences deposited in Affymetrix NetAffx™ Analysis Center (http://www.affymetrix.com/analysis/index.affx) mapped the 5 probesets to exons 1 to 4 from the Affymetrix Human Genome U133 Plus 2.0 Arrays used in the analysis onto specific *IKZF1* exons visualized using the UCSC genome browser (http://genome.ucsc.edu/cgi-bin/hgBlat?command=start). Examination of the Affymetrix probeset coverage in relation to most common *IKZF1* microdeletions observed in Ph^+^ BPL cases showed that all of these deletions can be detected by multiple *IKZF1* probesets. The most common microdeletion occurs between Exons 4-7 (30%) [14] that would be detected by 1565816_at and 1565818_s_at followed by Exons 2-7 (15%) [14] that would be detected by 1565816_at, 1565818_s_at, 220704_at, 1557632_at). The Mixed Model ANOVA explained 84.2% of the variation in the gene expression data across all 5 *IKZF1* transcripts covering Exons 1-4 (31% variation from the random factor for individual cases and 69% from the fixed factors (significant effects of Probeset (F_4,2084_ = 1828, P<0.0001), Diagnosis (F_2,521_ = 10.9, P<0.0001) and the Interaction term (F_8,2084_ = 9.1, P<0.0001) were observed for these fixed factors)). Our analysis demonstrated no changes in expression that would be expected from homozygous or heterozygous deletions of *IKZF1* in primary leukemic cells (Figure **3A**). In particular, the probesets 1565816_at (specific for Exons 1-4) and 1565818_s_at (specific for Exon 4 only) did not detect any significantly reduced expression levels in Ph^+^ cases comparisons versus Normal or Ph^-^ cases (Figure **3B**) controlling for repeated measures taken from individual cases, and were highly correlated across samples (Figure **3C**). Contrary to expectation, both probesets for Exon 3 exhibited significant increases in expression for Ph^+^ cases compared to Ph^-^ cases (Figure **3B**). Taken together, this expression data do not show reductions in levels for Ph^+^ cases that would have suggested (1) homozygous or heterozygous whole *IKZF1* locus deletions or (2) intragenic *IKZF1* deletions between Exons 4-7 or Exons 2-7.

### Constitutive Function of Ikaros in Leukemia Cells from Patients with Newly Diagnosed Pediatric High Risk BPL, Including Its Ph^+^ Subset

Transcript levels for validated IK target genes with documented IK binding sites that are upregulated by IK and downregulated in IK-deficient mice were highly correlated forming a subcluster in the hierarchical cluster representation (**Figures S1-S3 **in File **S1**). Forty-two transcripts representing 29 genes out of the 45 validated IK target genes harboring IK binding sites were up-regulated in 390 “high *IKZF1* expression” cases compared to 407 “low *IKZF1* expression” cases (**Figure S1 **in File **S1**). Expression profiles for *IKZF1* and these 42 IK target gene transcripts were highly correlated forming a subcluster in the hierarchical cluster representation. We examined the transcript levels and transcription factor function of native IK in primary ALL blast cells in diagnostic leukemic bone marrow samples from 576 B-lineage ALL/BPL and 174 T-lineage ALL patients. 21 of 42 (50%) transcripts representing 14 of 29 (48%) validated human IK target genes were expressed at markedly higher levels in leukemic bone marrow specimens from B-lineage ALL/BPL patients, including most of the patients with BCR-ABL ^+^ /Ph^+^ ALL (Figure **S2** in File **S1**; MANOVA, F_1,748_ = 168.7, P<0.0001). Similar results were obtained on diagnostic samples from two independent studies on 59 B-lineage ALL/BPL and 17 T-lineage ALL patients who subsequently experienced a relapse (Figure **S3** in File **S1**; MANOVA, Sum Contrast, F_1,74_ = 9.32, P=0.0032).

The transcripts of the IK target genes were not down-regulated in primary leukemia specimens of 122 BCR-ABL^+^ pediatric BPL patients vs. normal hematopoietic cells in 74 healthy bone marrow specimens reported in the “Microarray Innovations in Leukemia” (MILE) study (**Figure S4 **in File **S1**). A multivariate analysis of IK target gene expression levels showed that the multivariate mean for primary cells from BCR-ABL^+^ ALL patients was not significantly different from that of normal bone marrow cells (MANOVA, Sum Contrast, F_1, 194_ = 0.768, P=0.382). Thus, when compared to normal bone marrow specimens, the transcription factor function of IK, as measured by transcript expression levels of IK target genes, was not diminished in the leukemic bone marrow specimens from BCR-ABL^+^ pediatric BPL patients.

**Figure 4:**
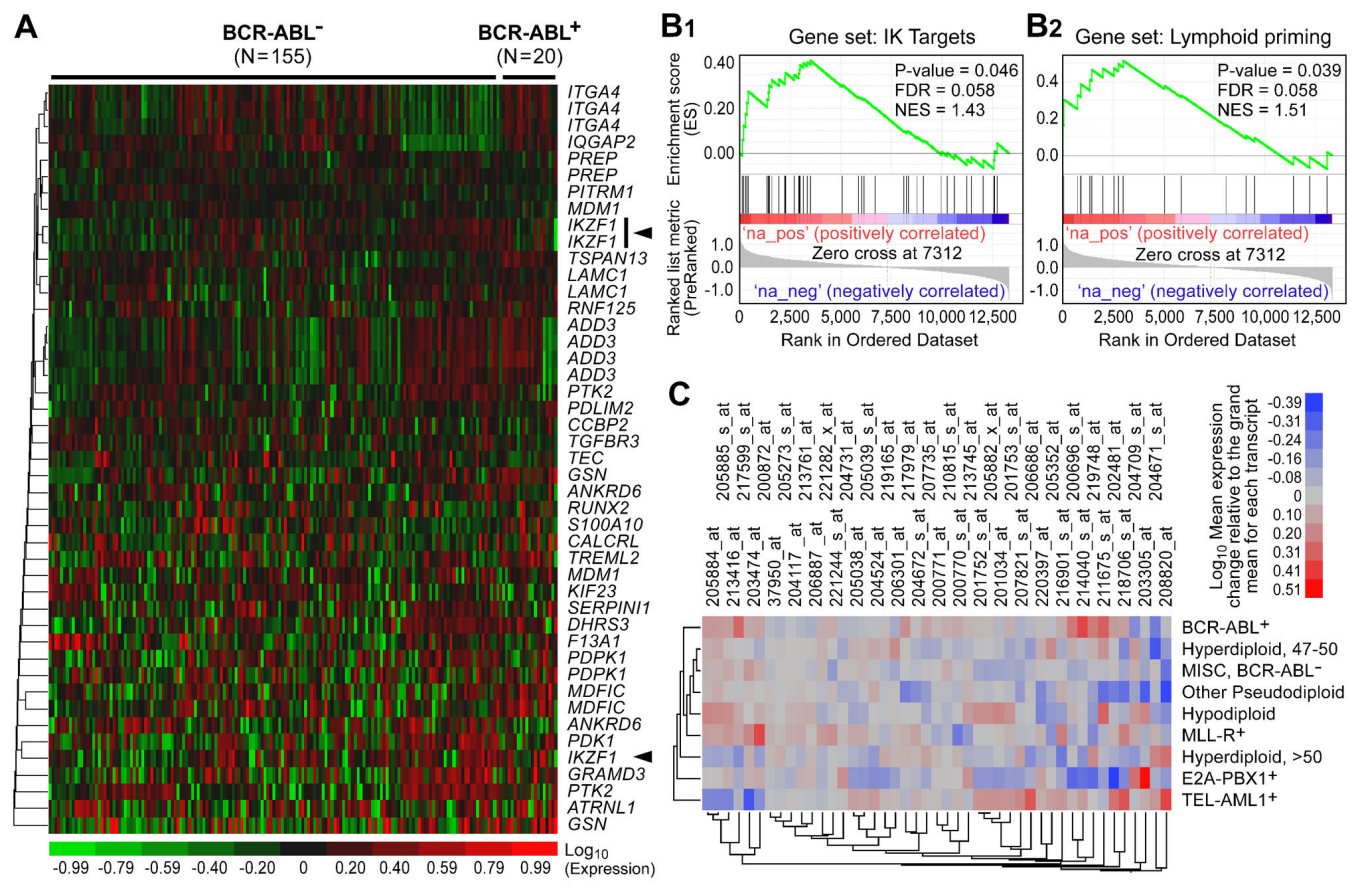
Transcript Levels of Ikaros Target Genes in Primary Leukemia Cells from Pediatric Ph^-^ and Ph^+^ BPL Patients. Expression levels of IK target genes were compared for primary leukemic cells from 155 pediatric Ph^-^/BCR-ABL^-^ BPL patients and 20 Ph ^+^ /BCR-ABL ^+^ /BPL patients on the Mullighan study (GSE12995). Transcript signal values were obtained from hybridization onto the Affymetrix Human Genome U133A genechip arrays. Heat map depicts up and down regulated transcripts ranging from red to green respectively for mean centered log_10_ transformed expression values and clustered according to average distance metric (**A**). Rank ordered difference in standard deviation units for Ph^+^ samples (N=20) compared to other samples (N=155) in the Mullighan study (GSE12995) were processed for enrichment of IK target genes **(B1)** and IK-regulated lymphoid priming genes **(B2)** using a supervised approach implemented in GSEA2.08 (Broad institute). Enrichment scores for calculated for the ranked members of the gene sets and normalized to the gene set size (NES) for which the P-value was calculated using 1000 permutations of the pre-ranked gene list and the FDR corrected for testing 2 gene sets. There was a significant enrichment of IK target genes (NES = 1.43, P = 0.046) for Ph^+^ patients that included the leading edge subset comprised of TSPAN13*, GSN*, MDFIC*, ITGA4, TREML2, RNF125*, IQGAP2, LAMC1, TES, DHRS3*, S100A10*, IL12RB1, ADD3*, GRAMD3, ATRNL1*, RUNX2*, MCOLN3*, ATP1B1, and CALCRL. Likewise, there was a significant enrichment of lymphoid priming genes (NES = 1.51, P = 0.039) for Ph^+^ patients that included the leading edge subset comprised of *IGJ*, CNN3, CD52, DNTT*, CSF1R*, SATB1*, LTB, PTGER2*, MEF2C, RUNX2 (Figure **1B.2**). There was a significant increase in the multivariate mean for 45 transcripts in the Ph^+^ subset of specimens compared to the pooled mean of the other subsets (MANOVA, F_1,173_ = 11.29, P=0.001). The mean level of expression for each transcript in each BPL subset is illustrated in the heat map organized using a two-way hierarchical clustering method (average distance metric) to group expression profiles of transcripts and specimen subsets (**C**).

**Figure 5:**
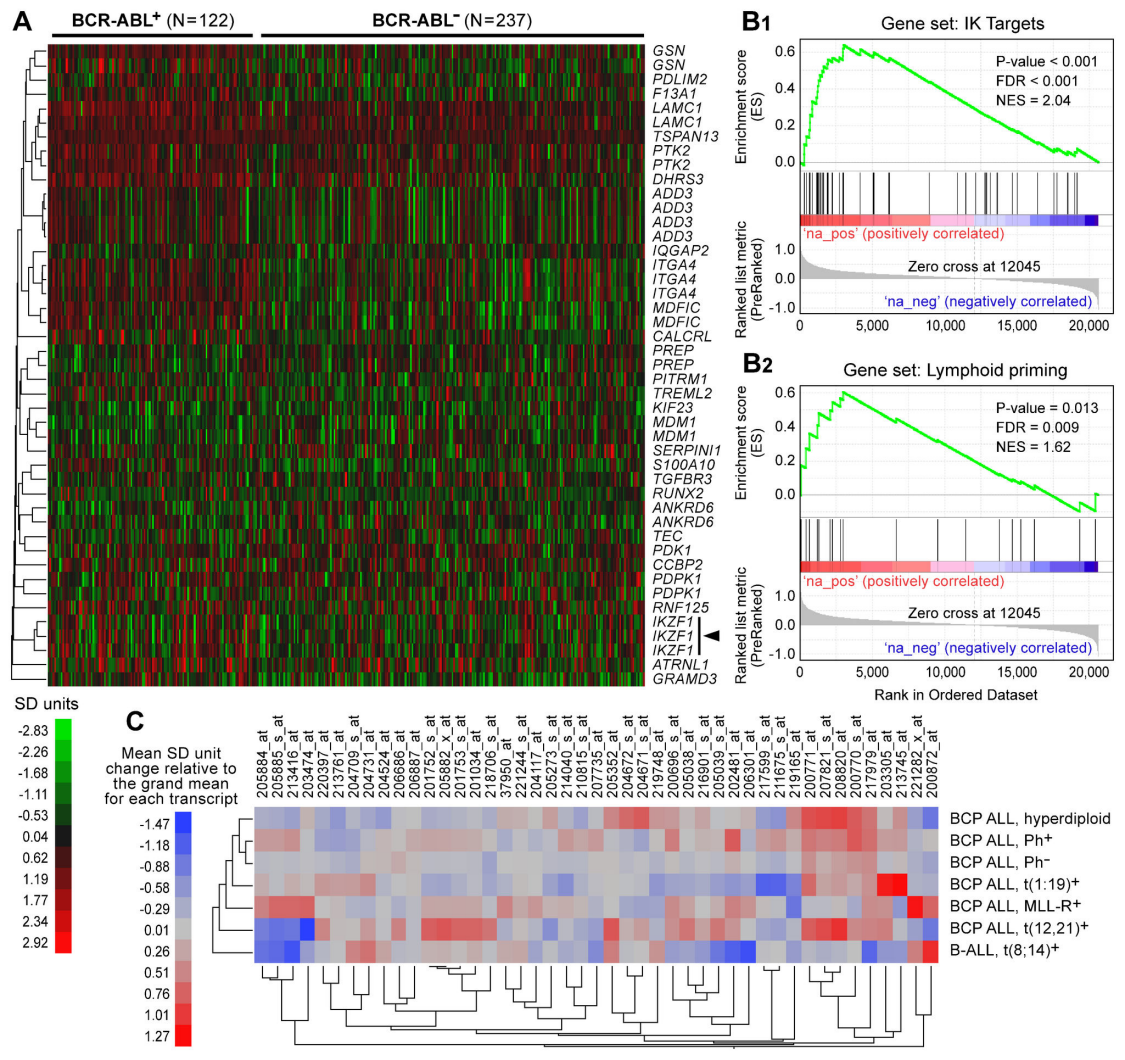
Transcript Levels of Ikaros Target Genes in Primary Leukemia Cells from Pediatric Ph^-^/BCR-ABL^-^ and Ph ^+^ /BCR-ABL^+^ BPL Patients on the MILE Study. Expression levels of IK target genes were compared between primary leukemic cells (GSE13159) from 122 pediatric BPL patients with t(9;22) translocation (Ph ^+^ /BCR-ABL^+^) and 237 pediatric BPL patients without t(9;22) translocation (Ph^-^/BCR-ABL^-^). Heat map depicts up and down regulated transcripts ranging from red to green respectively for standardized expression values and clustered according to average distance metric (**A**). 25 transcripts representing 16 IK target genes were expressed at significantly higher levels in leukemia cells from Ph^+^ patients, no significant differences were observed for 10 transcripts representing 9 IK target genes and 7 transcripts representing 5 IK target genes were significantly down regulated (**Table S2** in File **S1**). Hierarchical cluster analysis identified MDFIC (0.69 SD units, P = 8.8 x 10^-12^), DHRS3 (0.6 SD units, P = 3.7 x 10^-11^), *GSN* (0.49 SD units, P = 3.4 x 10^-10^), *ITGA4* (0.53 SD units, P = 2.6 x 10^-9^) and TSPAN13 (0.2 SD units, P = 1.2 x 10^-7^) as the most significantly up-regulated genes in the 122 Ph^+^ patient samples. Rank ordered difference in standard deviation units for Ph^+^ samples compared to Ph^-^ samples for enrichment of IK target genes **(B1)** as well as lymphoid priming genes **(B2)** using a supervised approach implemented in GSEA2.08 (Broad institute). Enrichment scores were calculated for the ranked members of the gene sets and normalized to the gene set size (NES) for which the p-value was calculated using 1000 permutations of the pre-ranked gene list and the FDR corrected for testing 2 gene sets. There was a significant enrichment of IK target genes (NES = 2.04, P < 0.001) and lymphoid priming genes (NES = 1.62, P = 0.013) that included the leading edge subsets MDFIC*, EIF4E3*, DHRS3*, ITGA4*, PTK2*, GSN, S100A10, GRAMD3*, F13A1, CALCRL, ATP1B1, LAMC1, TES, ADD3*, TCTEX1D1, ATRNL1*, IL12RB1*, RNF125, MCOLN3*, RUNX2, PDLIM2*, TSGA10, TREML2* for IK targets and *IGJ*, CNN3*, CSF1R, PTGER2, LTB*, DNTT, CD52, MEF2C, and RUNX2 for lymphoid priming genes. There was a significant increase in the multivariate mean for 45 transcripts in the Ph^+^ subset of specimens (MANOVA, F_1,357_ = 30.65, P<0.0001). The mean level of expression for each transcript in each BPL subset is illustrated in the heat map organized using a two-way hierarchical clustering method (average distance metric) to group expression profiles of transcripts and BPL subsets (**C**).

We next compared the transcript levels for IK target genes in primary ALL cells from 155 BCR-ABL^-^ and 20 BCR-ABL^+^ patients with high risk BPL. Of the 42 transcripts representing the IK target genes, only 3 transcripts were down regulated in BCR-ABL^+^ ALL cells (**Figure 4, Table S1 **in File **S1**). GSEA showed no evidence for down regulation of IK target genes in the BCR-ABL^+^ subset (**Figure 4B.1**). IK has been shown to direct lymphoid priming during the earliest stages of lymphocyte ontogeny by upregulating the expression of specific genes [17-19]. Of the 20 IK-regulated lymphoid priming genes, only two were down regulated in Ph^+^ leukemia cells (**Figure S5 **in File **S1**). A multivariate analysis of lymphoid priming gene expression levels showed that the multivariate mean for primary cells from Ph^+^ BPL patients was significantly higher than that of primary leukemia cells from Ph^-^ patients (MANOVA, Sum Contrast, F_1, 173_ = 7.04, P=0.0087). GSEA showed no evidence for down regulation of IK-regulated lymphoid priming genes in the Ph^+^ subset (**Figure 4B.2**). A multivariate analysis of IK target gene expression levels in cytogenetically distinct subsets of BPL showed that the multivariate mean for the Ph^+^ subset was significantly higher than all other groups combined (MANOVA, Sum Contrast, F_1,173_ = 11.29, P=0.001) (Figure **4C**). For validation of these findings, we next performed a bioinformatics analysis of the publically available international database of the MILE Study Group on gene expression profiles of primary leukemia cells from 122 Ph^+^ and 237 Ph^-^ pediatric BPL patients (Figure **5**). Of the 42 IK target transcripts representing 29 genes, only 7 transcripts representing 5 genes were significantly down regulated (**Figure 5A, Table S2 **in File **S1**). Of the 20 IK-regulated lymphoid priming genes, only 3 were down regulated in Ph^+^ leukemia cells (**Figure S6 **in File **S1**). GSEA showed evidence for up-regulation (not down-regulation) of IK target genes (P<0.001) (Figure **5B1**) and IK-regulated lymphoid priming genes (P=0.013) (Figure **5B2**) in the Ph^+^ subset. A multivariate analysis of IK target gene expression levels in cytogenetically distinct subsets of BPL showed that the multivariate mean for the Ph^+^ subset was significantly higher than all other B-precursor ALL groups combined (MANOVA, Sum Contrast, F_1,748_ = 168.7, P < 0.0001) (Figure **5C**). Taken together, these gene expression profiling data demonstrate that Ph^+^ ALL is not characterized by a functional deficiency of IK, as measured by expression levels of validated IK target genes.

**Figure 6:**
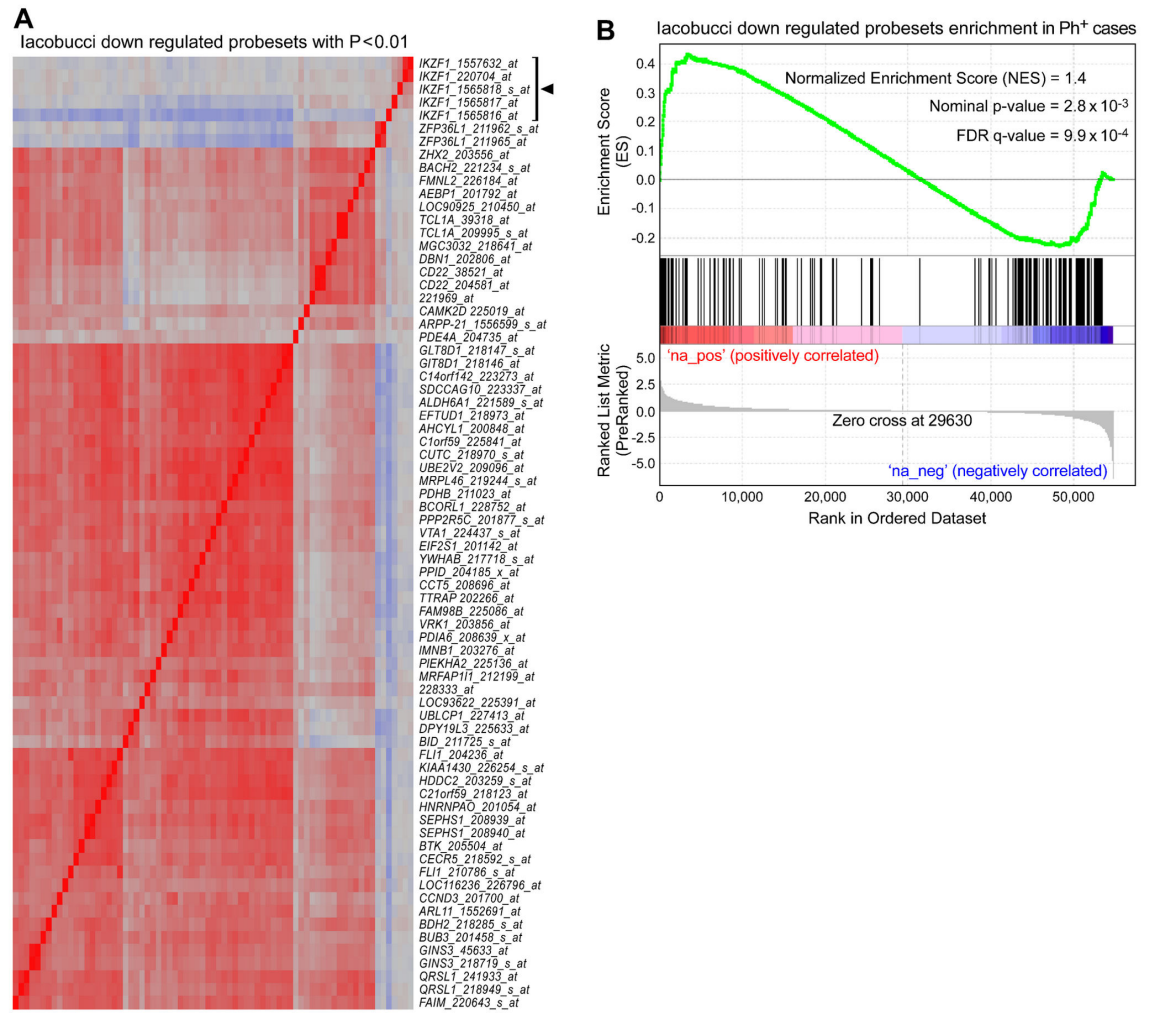
Gene Expression Signature for Adult IKZF1 Deletions Reported for Adult Ph^+^ BPL is not Found in Pediatric Ph^+^ BPL. [A] Correlation cluster forms distinct modules for expression of exon specific probe sets for IKZF1 and significantly down regulated probe sets for adult IKZF1 deletions in primary leukemic cells from pediatric BPL patients. Probeset correlations were clustered for the most significant down regulated probe sets in the Iacobucci study (P<0.01) and IKZF1 probesets specific for Exons 1-4 in pediatric leukemic samples. [B] Rank ordered difference in log_2_ transformed RMA expression values between primary leukemic bone marrow specimens from Ph^+^ BPL patients (N=123) and normal bone marrow specimens (N=74) were processed for enrichment of 158 Iacobucci down regulated probe sets using a supervised approach implemented in GSEA2.08 (Broad institute). Significance was assessed using weighted Kolmogorov-Smirnov statistics. Contrary to expectation, the gene signature for probe sets down regulated in IKZF1 deleted adult Ph^+^ BPL specimens were significantly over-represented in probe sets up regulated in Ph^+^ pediatric BPL cases (P-value = 2.8 x 10^-3^; hits shown by black lines on the graph and the heat map depicting blue color to indicate down regulation and red color to indicate up regulation of probesets in Ph^+^ BPL cases versus Normal).

Our findings are different from the results reported by Iacobucci et al. for adult Ph^+^ BPL patients [20]. Specifically, Iacobucci et al. reported that a group of IK-binding genes represented by 158 transcripts are downregulated in adult Ph^+^ BPL cells with heterozygous *IKZF1* deletions. Counterintuitively, *IKZF1* itself was not on the *Iacobucci* list of genes with reduced transcript levels in adult Ph^+^ BPL patients [20]. We examined 5 *IKZF1* probe sets specific for exons 1-4 and the 158 probeset *Iacobucci* signature for *IKZF1* deletions (as listed in Table S2 of reference [20]) for their co-regulation in pediatric BPL. Pairwise correlations and clustering of the correlation r-values revealed that the *IKZF1* probesets formed a distinct module compared to the *Iacobucci* gene signature (**Figure S7A **in File **S1**). Likewise, the probe sets for the *Iacobucci* gene signature were correlated with each other and 92% of the correlations were positive with P<0.05. However, the probesets for the *Iacobucci* genes did not exhibit the expected positive correlations with the IKZF1 probesets interrogating exons 1-4 and 42% of the correlations were negative with P<0.05 while 54% of the correlations did not show a statistical significance. This modular expression profile was also observed with the genes identified to contain *IKZF1* binding sites in mice (**Figure S7B **in File **S1**) as well as the most significantly down regulated Iacobucci gene set (Figure **6A**). We next cross-referenced the 158 probeset *Iacobucci* signature with gene expression changes in wild type vs. IKZF1 knockout mice and the presence of *IKZF1* binding sites from the archived CHiPseq data (GSM803110) [8]. None of the 29 genes from the *Iacobucci* gene set that exhibited *IKZF1* binding sites were down regulated in *IKZF1* knockout (“null”) mice [8]. A Gene Set Enrichment Analysis (GSEA) showed no significant enrichment of the downregulated transcripts of the *Iacobucci* probeset in primary leukemic specimens from Ph^+^ pediatric BPL patients compared to normal bone marrow specimens (Figure **6B**). In fact, Ph^+^ patients exhibited a significant over-representation of upregulated probesets (P = 2.8 x 10^-3^) that included the leading edge probesets comprised of *AEBP1, AFF3, BACH2, BCL2, BDH2, BLK, CD22, DBN1, DKFZP761P0423, ERGIC1, FADS3, FAIM, FLJ32997, FMNL2, HS6ST2, KIAA0802, LOC90925, MAG, MARCKSL1, MGC3032, MSH6, NULL, P4HA2, PCDH9, QRSL1, SMARCC1, TBC1D4, TCL1A, ZHX2*). These results demonstrate that the genes reported as a downregulated geneset for adult Ph^+^ BPL patients by *Iacobucci* are not downregulated in pediatric Ph^+^ BPL patients. The lack of a correlation between the expression levels of these genes and IK expression levels in adult Ph^+^ BPL patients, pediatric Ph^+^ BPL patients, or mice suggest that they are not likely to be genes selectively regulated by IK. The lack of a strong correlation of the *IKZF1* probesets with the *Iacobucci* probeset led us to examine other potential transcription factors that could modulate the expression of these IK-binding but not IK-regulated genes. Specifically, we interrogated the *Iacobucci* gene signature with the Molecular Signatures Database (http://www.broadinstitute.org/gsea/msigdb/index.jsp) for over representation of transcription factor binding motifs. Significantly over-represented motifs included the binding motif for several transcription factors, including (1) MYC (“CACGTG”; *ALDH18A1, ALDH6A1, CAMK2D, CBX5, CLTC, EEF1E1, FADS3, GCSH, LMNB1, PFDN6, PLCG2, SORD, SUPT16H, ZHX2*; FDR q-value=0.000423) (2), OCT1 (“MKVATTTGCATATT”; *BCL2, BTK, CDCA7, YWHAB, ZFP36L1, ZHX2*; FDR q-value = 0.0196) (3), SREBF1 (“TCANNTGAY”; *AHCYL1, ALDH6A1, CAMK2D, CAPN3, EIF2S1, SUPT16H, VPS35*; FDR q-value = 0.026), and (4) E2F1 (“TTTSGCGS”; *ALDH6A1, CDCA7, FLI1, GINS3, MSH2*; FDR q-value = 0.026).

### Constitutive Function of Ikaros in Relapse Clones and Xenograft Cells from Pediatric BPL Patients

Gene expression profiles of primary leukemia cells in diagnostic bone marrow specimens of BPL patients who subsequently experienced an early relapse (N=40; time to relapse <36 months) were not suggestive of diminished IK activity when compared to primary leukemia cells from patients who experience a late relapse (N=19; time to relapse ≥36 months) (**Figure S8 **in File **S1**). There was a significant increase in the multivariate mean for 45 transcripts (3 transcripts for IKZF1 and 42 transcripts for 29 IK target genes) comparing Early to Late Relapse subsets (Average fold increase = 1.3; MANOVA, F1,57 = 7.11, P=0.01). This finding suggested that high (rather than low) blast cell IK activity may be a biomarker for subpopulations of patients who are at high risk for treatment failure and early relapse.

Gene expression profiling of primary leukemic cells from matched pair relapse vs. diagnostic bone marrow specimens of 59 patients with BPL who relapsed showed similar expression levels for IK target genes (**Figure S9 and Table S4 **in File **S1**) (4 transcripts for 4 genes exhibited significant up-regulation of IK targets *KIF23*, *ANKRD6*, *ATRNL1* and *LAMC1* with no significant differences for the remaining IK targets). Since development of aggressive leukemia in mice with heterozygous deletions of the IK exons encoding the zinc fingers required for sequence-specific DNA binding occurs only with a concomitant loss of heterozygosity between 3 and 6 months after birth [19], we sought to determine if leukemic blast cells from aggressive early relapse patients are characterized by homozygous deletions of *IKZF1* exons 4-7 involved in reported heterozygous IK deletions in high-risk BPL. To this end, we performed exon-specific PCR with DNA sequencing on purified genomic DNA samples from 1^st^ relapse bone marrow specimens of 9 BPL patients who experienced an early relapse. As shown in Figure **S9B** in File **S1**, no *IKZF1* deletions involving E4-E7 were found in any of the 9 cases. Further, no mutations were detected in IKZF1 E4-E7 or adjacent intronic segments. We next used Western blot analysis with an IK-specific antibody to study IK protein expression. Leukemic cells from 9 of 9 relapsed BPL cases studied expressed a ~57-kDa immunoreactive protein corresponding in size to full-length IK1 (**Figure S9C **in File **S1**). These results do not suggest that IK deficiency contributes to progression of leukemia or emergence of resistant relapse clones in pediatric BPL.

We also examined the expression of IK in very aggressive *in vivo* clonogenic human leukemic lymphocyte precursor cells isolated from spleens of xenografted NOD/SCID mice that developed overt leukemia after inoculation with primary leukemic cells from 7 patients with B-precursor ALL. We recently reported that these cells are characterized co-expression of multiple ALL-associated human lymphoid differentiation antigens, including high levels of CD10, CD19, and CD34 that have been reported as markers of putative leukemic stem cells in B-precursor ALL [11]. Xenograft cells from all 7 cases expressed a ~57-kDa immunoreactive protein corresponding in size to full-length IK1 (**Figure S10 **in File **S1**). Smaller IK proteins of <47-kDa size (i.e., size of full-length IK2 and IK3) possibly corresponding to dominant negative IK isoforms were also detectable in 2 of the 7 cases. IK expression in ALL xenograft cells from 4 separate BPL cases was also confirmed by using an intracellular flow cytometry method (**Figure S10 **in File **S1** **, Panel B**). Despite the non-specific staining caused by the secondary antibody alone with this assay platform that employs an aqueous buffered solution containing formaldehyde and proprietary ingredients (BD Phospholow Fix Buffer 1, Cat. #: 557870, BD Biosciences, San Jose, CA) for permeabilization of cell membrane, in each of these 4 cases (**Figure S10 **in File **S1** **, Panels B1-B4**), IK expression was confirmed by a marked increase in mean-channel immunofluorescence when the anti-IK antibody was included in the staining procedure (**Figure S10 **in File **S1** **, Panel B5**). Evaluation of the xenograft cells for IK expression at the single cell level using high-resolution confocal microscopy confirmed nuclear and cytoplasmic expression of IK (**Figure S10C **in File **S1**). We also used real-time qPCR to examine side-by-side genomic DNA from normal hematopoietic cells in normal bone marrow specimens (N=2) and ALL xenograft cells (N=3) for evidence of intragenic deletions of *IKZF1* involving Exon 4. No xenograft case was found to have a significantly higher Ct value than normal controls suggestive of an *IKZF1* deletion (**Figure S10D **in File **S1**).

## Discussion

In the present study we examined the expression and function of IK protein in leukemic blast cells from children with BPL. To our knowledge, this is the first comprehensive analysis of IK function in primary leukemic cells from relapsed or newly diagnosed pediatric high-risk BPL patients. Our results demonstrate that IK deficiency is not a common characteristic of high-risk BPL or its Ph^+^ subset in children. In particular, gene expression profiling data from two independent studies showed that Ph^+^ ALL is not characterized by a functional deficiency of IK, as measured by expression levels of validated IK target genes with documented IK binding sites that are upregulated by IK and down-regulated in IK-deficient mice. Likewise, GSEAs revealed no evidence for downregulation of IK target genes or IK-regulated lymphoid priming genes in the Ph^+^ subset. A multivariate analysis of IK target gene expression levels in cytogenetically distinct subsets of BPL showed that the multivariate mean for the Ph^+^ subset was significantly higher than all other groups combined. Further, a group of IK-binding genes that were reported to be downregulated in adult Ph+ patients [20] were not downregulated in pediatric Ph^+^ BPL patients. We used real-time qPCR to examine side-by-side genomic DNA from normal hematopoietic cells in normal bone marrow specimens (and primary leukemic cells from 32 pediatric patients with high-risk BPL, including 4 Ph^+^ ALL patients, for evidence of intragenic deletions of *IKZF1*. No Ph^+^ or Ph^-^ high-risk BPL case was identified by interpatient comparisons or comparisons between leukemic and normal samples that had a significantly higher Ct value suggestive of an *IKZF1* deletion. Nuclear protein extracts from Ph^+^ and Ph^-^ pediatric BPL patients contained IK1, as determined by Western blot analysis, and exhibited IK-specific DNA binding activity in EMSAs. These results demonstrate that *IKZF1* deletions are not consistent abnormalities in pediatric high-risk BPL and they are not signature characteristics of pediatric Ph^+^ BPL, as previously reported.

In order to gain further insights into the incidence and biological significance of *IKZF1* deletions, we compared the expression levels of *IKZF1* transcripts in primary leukemic cells from pediatric Ph^-^ BPL patients in side-by-side comparison with pediatric Ph^+^ BPL cases and normal bone marrow specimens. The *IKZF1* probesets were mapped onto common *IKZF1* deletion regions to identify which probesets would be expected to exhibit reduced gene expression in samples with the reported deletions. Examination of the Affymetrix probeset coverage in relation to most common *IKZF1* deletions reported for B-cell precursor ALL cases showed that all of these deletions could be detected by multiple *IKZF1* probesets. The analysis demonstrated no changes in expression that would be expected from homozygous or heterozygous deletions of *IKZF1* in primary leukemic cells. In particular, the probesets 1565816_at (specific for Exons 1-4) and 1565818_s_at (specific for Exon 4 only) did not detect any significantly reduced expression levels in Ph^+^ positive or Ph- BPL vs. normal bone marrow specimens controlling for repeated measures taken from individual cases, that would have suggested heterozygous intragenic deletions between exons 4-7 or exons 2-7. Taken together with the IK function data presented here, these results indicate that in pediatric BPL, *IKZF1* deletions either occur in a minority of leukemic cells in a oligoclonal heterogeneous population of leukemic B-cell precursors or *IKZF1* expression is characterized by “allelic imbalance” or “allelic exclusion” and deletions occur in “inactive” alleles, as we recently proposed [21,22].

SNP arrays have been utilized to determine the copy number variations (CNV) for genes of interest in primary leukemia samples due to their ubiquitous genome coverage and the relatively high number of probes available to identify breakpoints during characterization of the CNV. However, despite the importance of CNV-disease association studies, CNV calling from SNP genotyping data is not well established. This is due to the fundamental limitation of SNP genotyping data for relating signal intensity to estimating the amount of DNA at a given locus resulting in concerns regarding the possibility of high false discovery (FDR) or low sensitivity rates for detecting CNVs [23-25]. Four main types of bias influence high FDR including cross-hybridizations, batch effects from varying data quality of SNP arrays, genomic waves of intensities induced by sequence-dependent hybridization rate and amplification efficiency. Most available algorithms do not address all the sources of high FDR for CNV determination and recent studies suggest multiple algorithms be used to achieve high confidence in DNA breakpoint estimation [26,27]. Less than 2% overlap was observed using 3 algorithms (PennCNV, QuantiSNP, and BirdSuite) to call CNVs from 10 samples chosen from the KARE study and 17% of the calls were common in overlaps of any 2 algorithms [26]. Validation of these CNVs revealed that only 38% could be verified by qPCR for calls made by a single algorithm and that this was increased to 71% for calls made by all 3 algorithms. Critical to understanding the prevalence of specific breakpoints across samples is the ability to statistically quantify variation in array quality that can result in high FDR. Most quality measurements provided by available software focus on single arrays and do not assess normalization across arrays to identify arrays with low signal to noise ratios or outlier distributions such as provided by gene expression arrays. The methodology to address this issue for SNP arrays has been published but is not widely used to accurately determine the prevalence of specific breakpoints in a population [28]. Furthermore, very little guidance is provided in software packages for array preprocessing, setting the parameters of signal detection, segmentation of data, signal levels arising from cross talk between alleles, setting sequence specific background signals with respect to optimizing sensitivity and specificity of CNV determination for each array and across multiple arrays. Therefore, to more accurately assess the prevalence of specific breakpoints for a given disease, experimental design must include technical replicates that can detect low quality arrays even when they could not be detected according to venders’ quality control (QC) suggestions [29] and include direct comparison to normal samples paired with disease samples to circumvent many of the statistical issues associated with setting parameters for single array segmentation methods, and relying on permutation p-values can be biased by outlier signals derived from very affinity probe binding and amplification differences [30,31]. The use of control datasets allows for the direct comparisons of noise in the cancer and normal samples and utilization of F- and T-statistics for the probes covering the same genomic regions offsetting artifacts such as genomic waves and intensity dependent noise levels. The statistics obtained from comparisons with normal samples also allow for direct comparisons of breakpoints from metaanalysis of multiple studies to gain stronger associations between CNV and disease.

While *IKZF1* deletions in hemizygous constellation have been reported to occur in BPL patients, we found no evidence to suggest that primary leukemic cells from either BCR-ABL^+^ or fusion negative BPL patients are IK-deficient based on protein expression levels or functional activity of IK. To the contrary, our study illustrates that IK is abundantly expressed and functional in primary BPL cells. Notably, relapse clones as well as very aggressive *in vivo* clonogenic leukemic B-cell precursors isolated from spleens of xenografted NOD/SCID mice that developed overt leukemia after inoculation with primary leukemic cells patients with BPL always expressed the IK1 protein. Therefore, the reported relationship between *IKZF1* deletions detected by SNP arrays and treatment outcome of pediatric high-risk BPL is likely to be a reflection of the genomic instability of aggressive leukemic clones rather than a phenomenon related to *IKZF1* haploinsufficiency, as originally proposed [3,4].

## Supporting Information

File S1 **Supporting information including materials and methods and supplemental figures/tables.** **Figure S1, Correlation Between Transcript Expression Levels of Ikaros Target Genes in Primary Leukemic Cells from Pediatric ALL Patients**. Expression values expressed as Standard Deviation units were compiled for the 7 studies and rank ordered according to the mean expression of three highly correlated transcripts (205038_at, 205039_s_at, 216901_s_at, 227344_at and 227346_at; 3 of these were common in all Affymetrix platforms - 205038_at, 205039_s_at, 216901_s_at). Heat map depicts up and down regulated transcripts ranging from red to green respectively and clustered according to average distance metric (A). T-tests were performed for the combined Standard Deviation units from the 5 datasets (2-sample, Unequal variance correction, P-values<0.05 deemed significant) to reveal 42 transcripts representing 29 genes as significantly up-regulated in samples with high *Ikaros/IKZF1* expression (B). **Figure S2, Ikaros Target Gene Transcript Levels in Primary Leukemic Cells from Diagnostic Bone Marrow Specimens of Newly Diagnosed Pediatric ALL Patients**. We compiled the archived “The Microarray Innovations in Leukemia” (MILE) study gene expression profiling (GEP) data on primary leukemic cells (GSE13159) from 576 B-lineage ALL/BPL and 174 T-lineage ALL patients. Heat map depicts up and down regulated transcripts ranging from red to green respectively and clustered according to average distance metric (A). A cluster of 8 IK target genes (GSN, DHRS3, LAMC1, PTK2, TSPAN13, F13A1, TGFBR3, PDK1) exhibited highly significant down regulation in T-lineage ALL. Differential expression levels of *IKZF1* gene (3 transcripts) and 29 IK target genes (42 transcripts) were compared in T-tests utilizing the DQN3 values (2-sample, Unequal variance correction, P-values<0.05 deemed significant). Gene expression values were transformed into standard deviation units and effect sizes were reported using differences standard deviation units between B-lineage ALL and T-lineage ALL (B). There was a significant increase in the multi-variate mean of the standard deviation scores for these 45 transcripts comparing B-lineage ALL with T-lineage ALL (MANOVA, F_1,748_ = 168.7, P<0.0001). **Figure S3, Ikaros Target Gene Transcript Levels in Primary Leukemic Cells from Diagnostic Bone Marrow Specimens of Newly Diagnosed Pediatric ALL Patients who Subsequently Experienced a Relapse**. Expression levels of IK target genes were also examined for primary leukemic cells from matched-pair diagnosis vs. relapse specimens of B-lineage ALL/BPL patients using archived GEP datasets from 2 independent studies (GSE 3912, GSE18497). Gene expression values for leukemia cells obtained from 59 B-lineage ALL patients at diagnosis (GSE3912, N=32 and GSE18497, N=27) were compared to the gene expression values for leukemia cells obtained from 17 T-lineage ALL patients at diagnosis (GSE3912, N=3 and GSE18497, N=14). RMA-normalized values for the GSE18497 dataset and the MAS5- Signal intensity values for the GSE3912 dataset were log_10_ transformed and mean-centered to the average value for the diagnosis samples for each gene transcript in each study. Heat map depicts up and down regulated transcripts ranging from red to green respectively for expression values and clustered according to average distance metric (A). T-tests (2 sample) compared B- versus T-lineage ALL subsets for IK target gene expression levels in leukemic cells from initial diagnosis specimens of patients who subsequently experienced a relapse (B). There was a significant increase in the multi-variate mean for the 45 transcripts representing *IKZF1* gene (3 transcripts) and 29 IK target genes (42 transcripts) in B-lineage ALL compared with T-lineage ALL (MANOVA, F_1,74_ = 9.32, P=0.0032). **Figure S4, Ikaros Target Gene Transcript Levels in Primary Leukemia Cells from BCR-ABL+ B-precursor ALL (BPL) Patients Compared to Normal Bone Marrow Cells**. We compiled the archived “Microarray Innovations in Leukemia” (MILE) study gene expression profiling (GEP) data on primary leukemic cells (GSE13159) from 122 pediatric BCR-ABL^+^ B-precursor ALL (BPL) patients with t(9;22) translocation and normal hematopoietic cells from 74 non-leukemic bone marrow specimens. Differential expression levels of *IKZF1* gene (3 transcripts) and 29 IK target genes (42 transcripts) were compared in T-tests utilizing the DQN3 values (2-sample, Unequal variance correction, p-values<0.05 deemed significant). Gene expression values were transformed into standard deviation units calculated from the mean and standard deviation expression values for all the samples in each study and effect sizes were reported using differences standard deviation units between comparison groups. Heat map depicts up and down regulated transcripts ranging from red to green respectively for expression values and clustered according to average distance metric (A). Statistical differences were assessed utilizing MANOVA that showed no effect in the multivariate mean for the 45 transcripts in BCR-ABL^+^ BPL cells (F_1,94_ = 0.768, P = 0.382) and differences in the individual transcripts were reported using T-tests assuming unequal variances (B). Thirteen (7 genes) and 18 transcripts (15 genes) were up regulated and down regulated respectively (P<0.05). **Figure S5, Lymphoid Priming Gene Transcript Levels in Primary Leukemia Cells from Pediatric BCR-ABL- and BCR-ABL+ BPL Patients**. Expression levels of lymphoid priming gene transcripts were compared for primary leukemic cells from 155 pediatric BCR-ABL^-^ BPL patients vs. 20 BCR-ABL^+^ BPL patients on the Mullighan study (GSE12995). Transcript signal values were obtained from hybridization onto the Affymetrix Human Genome U133A gene chip arrays. Heat map depicts up and down regulated transcripts ranging from red to green respectively for mean centered log_10_ transformed expression values and clustered according to average distance metric. **Figure S6, Lymphoid Priming Gene Transcript Levels in Primary Leukemia Cells from BCR-ABL- and BCR-ABL+ Pediatric BPL Patients from the MILE Study**. Expression levels of lymphoid priming transcripts were compared in primary leukemic cells from BCR-ABL^-^ B-precursor ALL patients *vs*. BCR-ABL^+^ B-precursor ALL patients on MILE study (GSE13159). Transcript signal values were obtained from hybridization onto the Affymetrix Human Genome U133A genechip arrays. Heat map depicts up and down regulated transcripts ranging from red to green respectively for standardized expression values and clustered according to average distance metric. Multivariate means between these two groups was not significant (MANOVA, F_1,357_ = 1.664, P = 0.198). **Figure S7, Correlation Cluster of Gene Expression Profiles of Primary Leukemic Cells from Pediatric BPL Patients Forms Distinct Modules of Expression for IKZF1 vs**. **Iacobucci Signature Genes for Adult Ph+ BPL with IKZF1 Deletions**. [A] Pairwise correlations were performed using the adult gene signature for *IKZF1* deletions (158 probe sets down regulated in adult Ph^+^ BPL patients with *IKZF1* deletions and the 5 *IKZF1* probe sets for Exons 1-4 for pediatric BPL cases using the RMA normalized dataset (N=123 Ph^+^ cases and 327 Ph^-^ cases). Correlation coefficients (r) were determined between all gene pairs and cluster analysis was applied to the matrix of correlation coefficients for both rows and columns of probe set identifications whereby red indicated positive correlations between probe set pairs and blue indicated negative correlation between probe set pairs. The majority of correlations between the 158 probe sets were significantly positive in direction (92% with P<0.05 (11456/12403 pairs) (Median correlation coefficient = 0.43; Interquartile Range = 0.28 to 0.57), whereas the majority of correlations for each of the *IKZF1* probe sets with each of the 158 probe sets for the adult *IKZF1* deletion signature exhibited either a positive correlation (42% with P<0.05 [329/790 pairs]) or no correlation (54% with P>0.05 [428/790 pairs], Median correlation coefficient = -0.07, Interquartile Range = -0.16 to -0.01) in pediatric BPL cases. [B] Probeset correlations were clustered for a subset of 29 IK target genes that harbored IK binding sites in mice identified by cross-referencing this gene set with the archived CHiPseq data (GSM803110 archived in GSE32311 [3] and *IKZF1* probe sets specific for Exons 1-4 in pediatric BPL samples. **Figure S8, Ikaros target gene transcript levels in primary leukemia cells from newly diagnosed pediatric BPL patients who subsequently experience an early vs**. **late relapse**. Expression levels of IK target genes were examined for primary leukemic cells from matched-pair diagnosis *vs*. relapse specimens of BPL patients using archived GEP datasets from 2 independent studies (GSE 3912, GSE18497). IK target gene expression levels were compared for leukemic cells from initial diagnostic specimens of patients who subsequently experienced an early relapse (N=40; <36 months) versus a late relapse (N=19; >36months) (2-sample T-test). Heat map depicts up and down regulated transcripts ranging from red to green respectively for log_10_ transformed mean centered expression values and clustered according to average distance metric (A). T-tests (2 sample) compared for Early versus Late relapse subsets for IK target gene expression levels in leukemic cells from initial diagnosis specimens of these patients. (B) There was a significant increase in the multivariate mean for 45 transcripts (3 transcripts for IKZF1 and 42 transcripts for 29 IK target genes) comparing Early to Late Relapse subsets (Average fold increase = 1.3; MANOVA, F1,57 = 7.11, P=0.01). **Figure S9, Primary Leukemia Cells from Pediatric BPL Patients in Relapse are not Characterized by Ikaros Deficiency**. [A] Matched pair gene expression values for leukemia cells obtained from 59 BPL patients at diagnosis and then at relapse (combined from GSE3912, N=32 and GSE18497, N=27). RMA-normalized values for the GSE18497 dataset and the MAS5- Signal intensity values for the GSE3912 dataset were log_10_ transformed and mean-centered to the average value for the diagnosis samples for each gene transcript in each study. To determine the differential expression of each gene, paired T-tests were performed for the combined mean-centered values from GSE3910 and GSE18497 datasets (Unequal variance correction, P<0.05 deemed significant). Heat map depicts up and down regulated transcripts ranging from red to green respectively for standardized expression values and clustered according to average distance metric. [B] Exon-specific genomic PCR with DNA sequencing on purified genomic DNA samples from 9 patients with BPL in first bone marrow relapse that occurred within 12 months of the completion of primary therapy. *IKZF1* Exons E4-7 and their intron-exon junctions were PCR amplified, as described in Materials and Methods using the PCR primers listed in Table S3. Normal size PCR products were obtained in each of the 9 patients, providing strong evidence against the existence of homozygous deletions of the entire *IKZF1* locus or within *IKZF1* exons 4-7. [C] IK Western blot analysis on whole cell lysates was performed as described in Materials and Methods. See text for discussion. **Figure S10, BPL Xenograft Cells are not Ikaros-Deficient**. [A] IK Western blot analysis was performed on ALL xenograft cells (and RAJI cell line that was included as a positive control). Xenograft cells were CD10^+^CD19^+^CD34^+^HLA-DR^+^ and caused overt leukemia in NOD/SCID mice after reinjection [2]. [B] IK expression in BPL xenograft cells was also detected by intracellular flow cytometry. B1 = Xeno-1 in A; B2 = Xeno-2 in A; B3 = Xeno-3 in A; B4 = Xeno-12.2 in A. [C] Confocal fluorescence microscopy showed punctate nuclear and perinuclear localization of IK in xenograft cells from two separate cases (C1-C3 = Xeno-1 in A and C4-C6 = Xeno-5 in A). System Magnification: 630 (Magnification[objective]: 63 x Magnification[Eyepiece]: 10). [D] Shown in D1 are the mean Ct values for Exon 4 and Exon 7 amplicons obtained by qPCR of genomic DNA samples from 3 BPL xenograft cases (Xeno-1, Xeno-2, and Xeno-5 in A) and normal hematopoietic cells from 2 non-leukemic normal bone marrow specimens in two independent experiments. Depicted in D2 are the bar graphs of Ct data from [D1] for Exon 4-specific qPCR after normalization by referencing the raw data to the Ct values from Exon 7-specific qPCR. Depicted in D3 and D4 the are representative cycle number vs. Log (∆Rn) (fluorescence signal) amplification plots obtained using the *IKZF1* Exon 4-specific test primers and *IKZF1* Exon 7-specific reference primers respectively for the normal hematopoietic cells and the 3 BPL xenograft cases. **Table S1, Expression Levels of Ikaros Target Genes in Ph(BCR-ABL) + vs. Ph(BCR-ABL**)**- BPL** **Patients – Mullighan** **Study**. Expression levels of IK target genes were compared in primary leukemic cells from 155 pediatric BCR-ABL^-^ BPL patients and 20 BCR-ABL^+^ BPL patients on the Mullighan study (GSE12995). Trimmed mean target intensity of each array was globally scaled to 500 (MAS5 values) as the normalization method. T-tests were performed using log_10_ transformed MAS5 signal values (2-sample, Unequal variance correction, p-values<0.05 deemed significant) to identify differentially regulated transcripts for IK target genes. **Table S2, Expression Levels of Ikaros Target Genes in BCR-ABL+ vs**. **BCR-ABL- BPL Patients – MILE Study**. Expression levels of IK target genes were compared in primary leukemic cells from 122 BCR-ABL+ and 237 BCR-ABL- pediatric BPL patients (MILE Study, GSE13159). Transcript signal values obtained from hybridization onto the Affymetrix Human Genome U133 Plus 2.0 Arrays were calculated using non-central trimmed mean of differences between perfect match and mismatch intensities with quantile normalization (DQN3, signal normalized with quartiles of the beta distribution with parameters p=1.2 and q=3. Of the 42 IK target transcripts representing 29 genes, only 7 transcripts representing 5 genes were significantly down regulated in BCR-ABL^+^ patients. 25 transcripts representing 16 genes were expressed at significantly higher levels in leukemia cells from BCR-ABL^+^ patients. Hierarchical cluster analysis identified *MDFIC* (0.69 SD units, P = 8.8 x 10^-12^), *DHRS3* (0.6 SD units, P = 3.7 x 10^-11^), *GSN* (0.49 SD units, P = 3.4 x 10^-10^), *ITGA4* (0.53 SD units, P = 2.6 x 10^-9^) and *TSPAN13* (0.2 SD units, P = 1.2 x 10^-7^) as the most significantly up-regulated genes in the 122 BCR-ABL^+^ patient samples. **Table S3, Primer Sets used for Amplifying and Sequencing Ikaros/IKZF1 Exons 4, 5, 6 and 7 and their Exon-Intron Junctions**. **Table S4, Expression Levels of IK Target Genes in Primary Leukemia Cells from Matched-Pair Relapse vs**. **Diagnosis Specimens of Pediatric BPL Patients**. Matched pair gene expression values of IK target genes for leukemia cells obtained from 59 BPL patients at diagnosis and then at relapse (combined from GSE3912, N=32 and GSE18497, N=27). RMA-normalized values for the GSE18497 dataset and the MAS5- Signal intensity values for the GSE3912 dataset were log_10_ transformed and mean-centered to the average value for the diagnosis samples for each gene transcript in each study. To determine the differential expression of each gene, paired T-tests were performed for the combined mean-centered values from GSE3910 and GSE18497 datasets (Unequal variance correction, P<0.05 deemed significant). We also compared the IK target gene expression levels in leukemic cells from initial diagnosis specimens of patients who subsequently experienced an early relapse (N=40; <36 months) versus a late relapse (N=19; >36months) (2-sample T-test).
(PDF)
