# Supplementary material for: Constitutive Function of the Ikaros Transcription Factor in Primary Leukemia Cells from Pediatric Newly Diagnosed High-Risk and Relapsed B-precursor ALL Patients
Source: PLoS One. 2013 Nov 20;8(11):e80732. doi: 10.1371/journal.pone.0080732 (PMC3835424; doi:10.1371/journal.pone.0080732)
Supplement: File S1 — Supporting information including materials and methods and supplemental figures/tables. Figure S1, Correlation Between Transcript Expression Levels of Ikaros Target Genes in Primary Leukemic Cells from Pediatric ALL Patients. Expression values expressed as Standard Deviation units were compiled for the 7 studies and rank ordered according to the mean expression of three highly correlated transcripts (205038_at, 205039_s_at, 216901_s_at, 227344_at and 227346_at; 3 of these were common in all Affymetrix platforms - 205038_at, 205039_s_at, 216901_s_at). Heat map depicts up and down regulated transcripts ranging from red to green respectively and clustered according to average distance metric (A). T-tests were performed for the combined Standard Deviation units from the 5 datasets (2-sample, Unequal variance correction, P-values<0.05 deemed significant) to reveal 42 transcripts representing 29 genes as significantly up-regulated in samples with high Ikaros/IKZF1 expression (B). Figure S2, Ikaros Target Gene Transcript Levels in Primary Leukemic Cells from Diagnostic Bone Marrow Specimens of Newly Diagnosed Pediatric ALL Patients. We compiled the archived “The Microarray Innovations in Leukemia” (MILE) study gene expression profiling (GEP) data on primary leukemic cells (GSE13159) from 576 B-lineage ALL/BPL and 174 T-lineage ALL patients. Heat map depicts up and down regulated transcripts ranging from red to green respectively and clustered according to average distance metric (A). A cluster of 8 IK target genes (GSN, DHRS3, LAMC1, PTK2, TSPAN13, F13A1, TGFBR3, PDK1) exhibited highly significant down regulation in T-lineage ALL. Differential expression levels of IKZF1 gene (3 transcripts) and 29 IK target genes (42 transcripts) were compared in T-tests utilizing the DQN3 values (2-sample, Unequal variance correction, P-values<0.05 deemed significant). Gene expression values were transformed into standard deviation units and effect sizes were reported using differences st [file pone.0080732.s001.pdf]

Supplemental Materials and Methods

Cells

Cryopreserved leukemia cells from children with newly diagnosed high risk B-precursor acute lymphoblastic leukemia (BPL) as well as children with BPL in first bone marrow relapse occurring within 12 months of the completion of primary therapy were examined for IK expression using multiple assay platforms. We also used BPL cells isolated from spleen specimens of xenografted NOD/SCID mice in the described experiments. The xenografts were established using primary cells from pediatric BPL patients. The IRB (CCI) at Children's Hospital Los Angeles (CHLA) (Human Subject Assurance Number: FWA0001914) determined that the use of leukemic cells in our research did not meet the definition of human subject research per 45 CFR 46.102 (d and f) since it does not include identifiable private information. The research was approved by the CHLA IRB/CCI. The IRB approved project numbers were CCI-09-00304 (CCI Review Date 12/21/2009, Approval Date: 12/29/09) for cryopreserved cells and CCI-10-00141 (CCI Review Date 7/27/2010, Approval Date 7/27/2010) for freshly obtained primary leukemia cells. We further used the ALL-1 (Ph^+ adult BPL, B-lineage), and RAJI (Burkitt's leukemia/lymphoma; ATCC®, CCL-86) cell lines.

Genomic PCR and Realtime Quantitative PCR Analysis of the *Ikaros/IKZF1* Gene in Leukemia Cells. DNA sequencing was carried out on primary leukemic cells from 3 Ph^+ pediatric high-risk BPL patients using a "primer-walking" strategy and the BigDye Terminator v.3.1 cycle sequencing kit (Applied Biosystems, Foster City, CA), as previously reported [1]. Total genomic DNA was extracted from patients' leukemia cells using the Qiagen DNeasy Blood & Tissue kit (Catalog No. 6950) according to the manufacturer's specifications. PCR products encompassing the *IKZF1* exons 4, 5, 6, 7 and their exon-intron junctions were amplified using

previously reported genomic PCR primer sets [1], [2]. PCR products were separated on 1% agarose gels and sized using the 1-Kb Plus DNA ladder from Invitrogen (Cat. No. 10787-018). The PCR products were cleaned using the Qiagen QIAquick PCR Purification Kit (Cat No. 28104) and submitted to the DNA Sequencing Facility of Genewize Inc (CA) using the corresponding forward primer and the Applied Biosystems' dye-based (BigDye V3.1™) DNA sequencing method. DNA sequencing was performed on an ABI 3730 DNA Analyzer using a long read protocol. Sequence obtained from each genomic PCR product was analyzed and aligned using SeqMan II contiguous alignment software in the LaserGene suite from DNASTAR Inc. and the MegAlign multisequence alignment software in comparison with the wild-type *IKZF1* sequence (NCBI Reference Sequence: NCBI Reference Sequence: NT_007819.17 Homo sapiens chromosome 7, Genome Reference Consortium Human Build 37 (GRCh37.p9) primary reference assembly, www.ncbi.nih.gov) [1], [2].

We performed realtime quantitative (q) PCR for exons 4–7 of *IKZF1* on genomic DNA samples isolated from primary leukemic cells of 32 high-risk pediatric BPL patients, including 4 Ph^+ BPL patients, and normal hematopoietic cells from 2 normal non-leukemic bone marrow samples. We used the Applied Biosystems 7900HT Fast Real-Time PCR System housed in the CHLA Stem Cell Core Facility for performing the real-time quantitative PCR in 96-well plates. The PCR reactions employed the Power SYBR® Green PCR Master Mix Kit (Invitrogen, Cat #4367659) following the manufacturer's recommendations. In brief, genomic DNA in 50 µL reaction volume per sample was amplified according to 2-step thermal cycling conditions, including initial Taq polymerase activation at 95°C x 10 min followed by 40 cycles of denaturation at 95°C x 30 sec, annealing/extension at 60°C x 1 min with the same primer sets listed used in standard genomic PCR. Each qPCR reaction comprised 25 µl 2× SYBR Green PCR Master Mix, forward and reverse primer at 100 nM (final concentration) for the reference Exon 7 primers and 100 nM (final concentration) for the Exon 4/Exon 5 test primers, 105 ng genomic DNA template and

sterile water up to a final volume of 50 µl. Quantification was based on the increased reporter fluorescence, which was measured and recorded using the sequence detection system. Results were expressed in terms of the threshold cycle value Ct; the cycle at which the change in fluorescence of the amplicon for the SYBR dye passes a significance threshold automatically determined by the real-time PCR system software (SDS2.3) based on the fluorescence of the fixed threshold no template control sample. Ct was identified as the intersection of the amplification curve and threshold line and used as a relative measurement of the concentration of the *IKZF1* gene template in the PCR reaction.

Reagents. Cell lines were propagated in RPMI 1640 cell culture medium (RPMI 16401X, Cat# 10-040-CV, Cellgro, Mediatech Inc, Manassas, VA) supplemented with 10% fetal bovine serum (Hyclone Characterized FBS Cat#: SH3007103PR, Thermo Fisher Scientific, Waltham, MA) and 1.0% penicillin/streptomycin (Cat# 15140-122, Gibco by Life Technologies, Carlsbad, CA). The rabbit polyclonal antibody for Ikaros (IK) 1 (H-100, sc-13039) was purchased from Santa-Cruz Biotechnology, Inc. (Santa Cruz, CA) for Western blot analysis of IK using previously reported procedures [1]. The mouse monoclonal anti-IK antibody used for confocal imaging was prepared in our laboratory [1]. Goat anti-mouse IgG: Horseradish Peroxidase (HRPO) (M15345) and goat anti-rabbit IgG: HRPO (R14745) antibodies were purchased from Transduction Labs. Green-fluorescent Alexa Fluor 488 dye-labeled secondary antibody Alexa Fluor 488 goat anti-mouse IgG (A-11001) for confocal microscopy was purchased from Invitrogen (Carlsbad, CA). UltraCruz™ Mounting Medium containing 1.5 µg/ml of 4', 6-diamidino-2-phenylindole (DAPI) was purchased from Santa-Cruz Biotechnology, Inc. (sc-24941). Molecular weight markers were purchased from Amersham Pharmacia Biotech. All chemicals used were reagent grade or higher. Restriction enzymes and proteinase inhibitors were purchased from Roche (Indianapolis, IN).

SCID Mouse Xenograft Model of Human BPL. We used an NOD/SCID mouse model of human B-precursor ALL [2]. NOD/SCID mice (NOD.CB17-*Prkdc*^{scid}/J; 4-6 weeks of age at the time of purchase, female) were obtained from the Jackson Laboratory (Sacramento, CA). The research was conducted according to Institutional Animal Care and Use Committee (IACUC) Protocol #280-09, that was approved by the IACUC of CHLA on 11-24-2009 and its 3-year rewrite application 280-12 that was approved on 7-10-2012. All animal care procedures conformed to the Guide for the Care and Use of Laboratory Animals (National Research Council, National Academy Press, Washington DC 1996, USA). NOD/SCID mice (6-8 week old, female, same age in all cohorts in each independent experiment) were inoculated with primary leukemic cells from patients with BPL by injecting 0.5-1x10^6 leukemia cells in 0.2 mL PBS i.v. via tail vein injection with a 27-gauge needle. Mice were monitored daily and electively euthanized at the indicated time points by CO_2 asphyxia. At the time of their death or elective sacrifice, mice were necropsied to confirm leukemia-associated marked splenomegaly. Spleens of mice were removed, sized, and cell suspensions were prepared for determination of mononuclear cell counts and immunophenotype. Leukemia cells isolated from spleens of xenografted mice in 7 different xenograft cases were examined for IK expression using Western blot analysis as well as confocal imaging and intracellular flow cytometry. The xenograft models and the immunophenotypic features of the All BPL xenograft cases used in the present study were recently reported [2].

Bioinformatics and Statistical Analysis of Gene Expression Profiles. The publically available archived GSE32311 database [3] was used to compare gene expression changes in control thymocytes from *IKZF1* wildtype mice (N=3; GSM800500, GSM800501, GSM800502) vs. IK-deficient thymocytes (N=8) from *IKZF1* null mice (N=3, GSM800503, GSM800504, GSM800505) with the same genetic background of (C57BL/6 x129S4/SvJae). Gene expression

changes were screened utilizing probe level RMA signal intensity values from the mouse 430_2.0 Genome Array to identify the gene signatures for up-regulated and down-regulated transcripts in *IKZF1* null mice by filtering changes greater than 2-fold and T-test P-values less than 0.05 (T-test, Unequal Variances, Excel formula). Application of this filter identified 1158 transcripts representing 924 genes that were down-regulated in *IKZF1* null mice with a subset of 201 transcripts representing 137 genes exhibiting >2-fold decreased expression levels. By cross-referencing this IK-regulated gene set with the archived CHiPseq data (GSM803110) using the Integrative Genomics Browser [4], we identified 45 IK target genes that harbored IK binding sites [1].

The Gene Pattern software (http://www.broadinstitute.org/cancer/software/genepattern) was utilized to extract expression values for human lymphocyte precursors from the National Center for Biotechnology Information (NCBI) Gene Expression Omnibus (GEO) database [5]. We compiled the archived gene expression profiling (GEP) data on 1486 primary leukemia specimens from pediatric ALL patients from 7 independent studies (GSE3912, N=113; GSE18497, N=82; GSE4698, N=60; GSE7440, N=99; GSE13159, N=750; GSE11877, N=207; GSE12995, N=175) that combined datasets from U133A and U133 Plus 2 genechips using common *IKZF1* probe sets to focus our analysis on 45 validated IK target genes as well as 20 lymphoid-priming genes [1]. For each study, the gene expression values were transformed into standard deviation units calculated from the mean and standard deviation expression values for all the samples in each study. Standardized values compiled from the 7 studies were rank ordered according to the mean expression of 3 highly correlated transcripts for *IKZF1* (205038_at, 205039_s_at and 216901_s_at). Consensus/exemplar sequences for two of the *IKZF1* probe sets (205038_at and 205039_s_at) exhibited alignment to exons 0 – 7 of the *IKZF1* sequence, and 1 probe set (216901_s_at) exhibited alignment to exons E2-E7 of the *IKZF1* sequence (UCSC genome browser alignment track utilizing blat followed by pslReps).

Prospective power analysis was utilized to determine the Standard Deviation cut-off for "high *IKZF1* expression" and "low *IKZF1* expression" samples in the data sets. We set the unadjusted critical P-value at $2.5x10^{-6}$ to control for False Positive Rate (FPR) at 0.05 in order to detect significant differences in any one of the *IKZF1* transcripts out of approximately 20,000 transcripts common across the 7 Affymetrix platforms. At this critical P-value, a total sample size greater than 254 would be sufficient to detect a difference of 1 standard deviation unit with 99.9% power for *IKZF1* transcripts. Therefore, samples were assigned to the "high *IKZF1* expression" group if their expression level was >0.5 standard deviation unit higher than the mean expression level (N=390) and to the "low *IKZF1* expression" group if their expression level was >0.5 standard deviation unit lower than the mean expression level (N=407). Forty-five validated IK target genes were represented by 60 transcripts common across 7 Affymetrix platforms. T-tests were performed using standardized expression values combined from 7 datasets (2-sample, Unequal variance correction, p-values<0.05 deemed significant). Forty-two transcripts representing 29 genes were up regulated in 390 "high *IKZF1*" samples compared to 407 "low *IKZF1*" samples. We used a one-way agglomerative hierarchical clustering technique to organize expression patterns using the average distance linkage method such that genes (rows) having similar expression across patients were grouped together (average distance metric). Dendrograms were drawn to illustrate similar gene-expression profiles from joining pairs of closely related gene expression profiles, whereby genes joined by short branch lengths showed the most similarity in expression profile across all samples.

Expression levels of IK target genes were also examined for primary leukemic cells from matched-pair diagnosis vs. relapse specimens of BPL patients using archived GEP datasets from 2 independent studies (GSE 3912, GSE18497 [6], [7]. Matched pair gene expression values for leukemia cells obtained from 59 BPL patients at diagnosis and then at relapse (combined from GSE3912, N=32 and GSE18497, N=27). RMA-normalized values for the

GSE18497 dataset and the MAS5- Signal intensity values for the GSE3912 dataset were $\log_{10}$ transformed and mean-centered to the average value for the diagnosis samples for each gene transcript in each study. To determine the differential expression of each gene, paired T-tests were performed for the combined mean-centered values from GSE3910 and GSE18497 datasets (Unequal variance correction, P<0.05 deemed significant). We also compared the IK target gene expression levels in leukemic cells from initial diagnosis specimens of patients who subsequently experienced an early relapse (N=40; <36 months) versus a late relapse (N=19; >36months) (2-sample T-test).

We compiled the archived "The Microarray Innovations in Leukemia" (MILE) study gene expression profiling (GEP) data on primary leukemic cells (GSE13159) from 122 pediatric Ph^+ BPL patients, 237 pediatric Ph^- BPL patients, 576 BPL, 174 T-lineage ALL and 74 normal bone marrow specimens [8]. Transcript signal values obtained from hybridization onto the Affymetrix Human Genome U133 Plus 2.0 Arrays were calculated using non-central trimmed mean of differences between perfect match and mismatch intensities with quantile normalization (DQN3, signal normalized with quantiles of the beta distribution with parameters p=1.2 and q=3 [9]. Differential expression of 45 IK target genes [1] were compared in T-tests utilizing the DQN3 values (2-sample, Unequal variance correction, p-values<0.05 deemed significant). Gene expression values were transformed into standard deviation units calculated from the mean and standard deviation expression values for all the samples in each study and effect sizes were reported using differences standard deviation units between comparison groups.

We also compared expression levels of IK target genes in primary leukemic cells from 155 pediatric $BCR\text{-}ABL^-$ BPL patients and 20 $BCR\text{-}ABL^+$ BPL patients on the Mullighan study (GSE12995) [10]. Transcript signal values were obtained from hybridization onto the Affymetrix Human Genome U133A genechip arrays. Trimmed mean target intensity of each array was

globally scaled to 500 (MAS5 values) as the normalization method. T-tests were performed using log_{10} transformed MAS5 signal values (2-sample, Unequal variance correction, p-values<0.05 deemed significant) to identify differentially regulated transcripts for IK target genes. Differential expression for a cassette of IK target genes was determined using Multivariate Analysis of Variance model utilizing mean- centered values to compare expression changes. This analysis compared differences in centroid values (multi-variate mean) between groups of samples being compared. Contribution of each transcript to the differences observed in multi-variate mean was assessed using Students T-tests.

Genes involved in lymphoid-lineage affiliated transcriptional program controlled by Ikaros. Twenty lineage-affiliated genes *(FLT3, NOTCH1, LTB, BTLA, CD52, CLNK, IL7R, CCR9, DNTT, IGJ, SATB1, SOX4, RUNX2, MEF2C, RAG1, HMGA2, CNN3, PTGER2, ETS1, CSF1R*) implicated in lymphoid priming were compiled from mouse studies for *evaluation* of their expression levels in lymphocyte precursors from primary leukemia specimens of children with ALL [1].

***IKZF1* Gene Expression Analysis Using Multiple Probe sets For Identification of *IKZF1* Deletions.** BLAT analysis on *IKZF1* target sequences deposited in Affymetrix NetAffx™ Analysis Center (http://www.affymetrix.com/analysis/index.affx) mapped these probe sets onto specific *IKZF1* exons visualized using the UCSC genome browser (http://genome.ucsc.edu/cgi-bin/hgBlat?command=start) [11]. This analysis is designed to locate sequences of 95% and greater similarity of length 25 bases or more in the entire genome [11]. The exon designation by comparing the BLAT analysis to 3 reference sequences (UCSC genes, Ensembl gene predictions, Human mRNA Genbank) were as follows: 1565817_at: Exon 1, chr7:50,348,436-50,348,502; 1565816_at: Exon 1-4 reverse strand, chr7:50,348,485-50,444,456; 1565818_s_at: Exon 4, chr7:50,444,230-50,444,480; 220704_at: Exon 3, chr7:50,368,140-50,368,398;

1557632_at: Exon 3, chr7:50,370,493-50,370,631; 216901_s_at: Exon 8, chr7:50,467,793-50,468,294; 205039_s_at: Exon 8, chr7:50,469,625-50,470,188; 205038_at: Exon 8, chr7:50,471,237-50,471,785; 227346_at: Exon 8, chr7:50,471,780-50,472,114; 227344_at: Exon 8, chr7:50,472,198-50,472,761.

We compiled 6 archived gene expression profiling datasets that measured expression from B-precursor ALL childhood patients hybridized to Human Genome U133 Plus 2.0 Array containing the 10 *IKZF1* probe sets for determination of exon specific expression (GSE11877, N=207; GSE13159 N=823; GSE13351 N=107; GSE18497, N=82, GSE28460, N=98; GSE7440, N=99; Total, N=1416). To enable comparison of samples across studies, a normalization procedure was performed that merged the raw data from the 6 datasets (CEL files). Perfect Match (PM) signal values for probe sets were extracted utilizing raw CEL files matched with probe identifiers obtained from the Affymetrix provided CDF file (HG-U133_Plus _ 2.cdf) implemented by Aroma Affymetrix statistical packages ran in R-studio environment (Version 0.97.551, R-studio Inc., running with R 3.01) [12]. The PM signals were quantified using Robust Multiarray Analysis (Bolstad 2003, Irizarry, 2003a 2003b) in a 3 step process including RMA background correction, quantile normalization, and summarization by Median Polish of probes in a probeset across 1416 samples (RMA method adapted in Aroma Affymetrix) [13]-[15]. RMA background correction estimates the background by a mixture model whereby the background signals are assumed to be normally distributed and the true signals are exponentially distributed. Normalization across all 6 studies and 1416 samples was achieved using a two-pass procedure. First the empirical target distribution was estimated by averaging the (ordered) signals over all arrays, followed by normalization of each array toward this target distribution.

A subset of pediatric BPL cases were further analyzed to compare leukemic specimens from Ph^+ BPL (N=122 from GSE13159 and N=1 from GSE13351) with leukemic specimens from Ph^-

BPL cases (N=236 from GSE13159 and N= 91 from GSE13351) as well as normal bone marrow specimens (N=74 from GSE13159) using 5 *IKZF1* probe sets that interrogated *IKZF1* exons 1-4. We performed a one-way hierarchical clustering technique to organize expression patterns (log_2 transformed RMA normalized values for 524 samples and mean centered to normal samples) such that transcripts (rows) with similar expression profiles across patients were grouped together using the average distance metric. Mixed Model Analysis of Variance analysis with three fixed factors ("Diagnosis" (Normal, Ph^-, Ph^+), "Probeset" (5 *IKZF1* probe sets), an interaction term for Diagnosis x Probeset) and a random factor, "case" for sample identification was utilized for the analysis of differential *IKZF1* gene expression levels. Planned Linear contrasts were performed using the fitted parameters from the interaction term to compare Ph^+ versus Ph^- BPL or normal samples for each of the 5 *IKZF1* probe sets. All calculations were performed using JMP statistical package (JMP v10, SAS, Cary, NC). We performed pairwise correlations of the genes (158 probe sets) reported by *Iacobucci* et al. to be down regulated in adult Ph^+ BPL patients with *IKZF1* deletions [16] and the 5 *IKZF1* probe sets for *IKZF1* Exons 1-4 in pediatric BPL patients using the RMA normalized database that combined 123 Ph^+ BPL cases and 327 Ph^- BPL cases. Correlation coefficients (r) were determined between all gene pairs and cluster analysis was applied to the matrix of correlation coefficients for both rows and columns of probeset identifications (JMP Software, SAS, Cary, NC). Modular structure of the probeset correlations was also deduced for the most significant down regulated probe sets in the *Iacobucci* study [16] (P<0.01), and for a subset of 29 IK target genes that harbored IK binding sites in mice identified by cross-referencing this gene set with the archived CHiPseq data (GSM803110 archived in GSE32311 [3] using the Integrative Genomics Browser [4].

Gene Set Enrichment Analysis (GSEA). Rank ordered differences in standard deviation units for BCR-ABL$^+$ BPL samples (N=20) compared to other samples (N=155) in the Mullighan study

(GSE12995) [10] and Ph$^+$ BPL (N=122) versus Ph- BPL (N=237) in the MILE study (GSE13159) [8] were processed for enrichment of lymphoid priming genes (18 genes represented on the gene chips) and IK target genes (39 genes represented in the Mullighan study, 45 genes represented in the MILE study) using a supervised approach implemented in GSEA v2.08 (Broad institute). These ranked ordered genes were screened for enrichment of gene sets in 13321 genes (22283 transcripts) for the Mullighan study and 20606 genes (54613 transcripts) for the MILE study using weighted Kolmogorov-Smirnov statistics implemented in GSEA (GSEA v2.08 (Broad Institute) [17]. Genes for multiple probes were collapsed using the "Max_probe" algorithm provided. GSEA evaluated significance of the over-representation of gene sets correlated or anti-correlated with expression in Ph$^+$ samples by calculating the Enrichment Score (ES) that represents the difference between the observed rankings from the expected null assuming a random rank distribution utilizing an empirical permutation test procedure that randomly assigned gene names to the rank ordered standard deviation values ("GSEA Pre-ranked" algorithm). Leading edge genes were identified up to and including the peak of the ES profile. Nominal P-values were computed by comparing the tails of the ES scores for observed and permutation-generated null distributions following 1000 permutations.

Rank ordered difference in $\log_2$ transformed RMA expression values between leukemic specimens from Ph$^+$ ALL patients (N=123) and normal bone marrow specimens ((N=74) from 2 studies (viz.: GSE13159 and GSE13351) were processed for enrichment of 158 probe sets that showed downregulation within the Ph$^+$ subset of the *Iacobucci* study [16] using a supervised approach implemented in GSEA2.08 (Broad institute). Significance was assessed using the weighted Kolmogorov-Smirnov statistics. GSEA evaluated significance of the over-representation of probe sets sets correlated or anti-correlated with *IKZF1* expression in Ph$^+$ samples by calculating the Enrichment Score (ES) representing the difference between the observed rankings from the expected null. The null distribution assumed a random rank

distribution utilizing an empirical permutation test procedure that randomly assigned probeset names to the rank ordered differences in expression ("GSEA Preranked" algorithm). Leading edge genes were identified up to and including the peak of the ES profile. Nominal P-values were computed by comparing the tails of the ES scores for observed and permutation-generated null distributions following 1000 permutations.

Western Blot Analysis of Ikaros Expression. Western blot analysis of whole cell lysates and nuclear protein extracts for IK expression was performed by immunoblotting using the ECL chemiluminescence detection system (Amersham Life Sciences), as described previously [1].

Electrophoretic Mobility Shift Assays (EMSAs). EMSAs were performed on nuclear extracts (NE) from BPL cells using the Thermo Scientific LightShift Chemiluminescent EMSA Kit (Catalog No. 20148) (Pierce, Rockford, IL, USA) following the manufacturer's protocol, as previously described [1], [18]. Preparation of nuclear extracts was carried out using the NE-PER Nuclear and Cytoplasmic Extraction Kit from Thermo Scientific (Pierce Protein Biology products, Catalog #78833). The protein concentrations of the extracts were determined with the standard A280 assay using a Thermo Scientific NanoDrop™ NanoDrop 1000 Spectrophotometer. Oligonucleotide probes for EMSA were purchased from Integrated DNA Technologies (IDT, San Diego, CA, USA) and included *IK-BS1* (5'-TCAGCTTTTGGGAATACCCTGTCA-3') containing a high-affinity IK1 binding site and *IK-BS5* (5'-TCAGCTTTTGAGAATACCCTGTCA-3') that has a single base pair (G>A) substitution at position 3 within the core consensus and does not bind IK [1], [18]. In these experiments, single stranded *IK-BS1* and *IK-BS5* oligonucleotides were biotin-labeled using the Biotin 3' End Labeling Kit (Catalog No. 89818) (Pierce, Rockford, IL, USA). 100 nmols of unlabeled oligonucleotides were incubated for 30 minutes at 37°C in a reaction mixture containing 1X TdT Reaction Buffer (500mM cacodylic acid, 10mM $CoCl_2$, 1mM DTT, pH 7.2), 0.5 μM Biotin-11-

UTP and 0.2 Units of TdT. 0.2 M EDTA was added to stop the reaction and biotin-labeled oligonucleotides were extracted using chloroform: isoamyl alcohol (24:1). Single-stranded biotinylated oligonucleotides were duplexed by mixing together equal amounts and incubating for 1 hour at room temperature. Each binding reaction for EMSA included 10X Binding Buffer (100 mM Tris, 500 mM KCl, 10 mM DTT), 2.5% Glycerol, 5 mM $MgCl_2$, 50 ng Poly (dI∗dC), 0.05% NP-40, 4 µg nuclear protein extract (NE), and 20 fmols of the biotin-labeled duplexed probe in a total volume of 20 µl. Binding reactions were performed at room temperature for 20 minutes. Supershift assays were performed with an anti-IK monoclonal antibody (2 µg/sample) to confirm the presence of IK in the retarded DNA-binding protein complexes, as previously described [18]. A 6% non-denaturing polyacrylamide gel was pre-run during the 20 min incubation time at 200 V in pre-chilled 0.5X TBE buffer (89 mM Tris base, 89 mM boric acid, 1 mM EDTA, pH ~8.0). 5X Loading Buffer was added to each reaction sample and samples were loaded onto a polyacrylamide gel. Samples were electrophoresed at 100V and transferred at 380 mA (~50 V) for 30 minutes to a Biodyne B Nylon Membrane (Catalog No. 77016) (Thermo Scientific, Rockford, IL, USA) soaked in 0.5X TBE buffer. When the transfer was complete, biotin-labeled DNA was cross-linked to the membrane at 120 mJ/cm^2 using a Spectrolinker XL-1000 UV cross-linker with 254-nm UV light bulbs. The biotin-labeled DNA was detected using a stabilized streptavidin-horseradish peroxidase (HRP) conjugate and a highly sensitive chemiluminescent substrate according to the manufacturer's instructions [1]. The membrane was exposed to X-ray film and developed with a film processor.

Immunofluorescence Staining and Multiparameter Flow Cytometry. A broad panel of commercially available monoclonal antibodies were used for screening immunophenotyping of primary leukemic cells from pediatric ALL patients as well as leukemic cells from spleen specimens of NOD/SCID mice xenografted with primary human ALL cells by two-color immunofluorescent staining and multiparameter flow cytometry as previously reported [2]. The

antibodies were purchased from BD Biosciences (San Jose, CA) and included: CD19-Phycoerythrin (PE) (Cat. #555413), CD7-Fluorescein isothiocyanate (FITC) (Cat. #555360), CD10-PE (Cat. #555375), HLA/DR/DP/DQ-FITC (Cat. #: 555558), CD45-PE (Cat. #: 555483), CD5-PE (Cat. #: 555354), CD34-FITC (Cat. #555821), CD33-FITC (Cat. #: 340533), and HLA-A, B, C-PE (Cat#555553). The IK expression level of leukemic cells was examined by intracellular flow cytometry using BD Phospholow Fix Buffer 1 (Cat. #: 557870, BD Biosciences, San Jose, CA) for permeabilization of the cell membrane [2] and indirect immunofluorescent staining with our mouse monoclonal anti-IK antibody [1] and the goat-anti-mouse-FITC secondary antibody (BD Biosciences Cat. #: 555988, San Jose, CA). The labeled cells were analyzed on a LSR II flow cytometer (Becton Dickinson, Lakes, NJ).

Confocal Laser Scanning Microscopy. Subcellular IK localization studies using immunofluorescence and spinning disk confocal microscopy were performed as previously described [1]. Slides were imaged using the PerkinElmer Spinning Disc Confocal Microscope and the PerkinElmer UltraView ERS software (Shelton, CT) or the Velocity V5.4 imaging software (PerkinElmer, Shelton, CT) [1]. The coverslips were fixed with ice-cold MeOH at -20 C for 10 minutes. The fixed cells were permeabilized and their non-specific antibody binding sites blocked with 0.1% Triton X-100 and 10% goat serum in PBS for 30 minutes, respectively. In order to detect and localize the IK protein, cells were stained with an in house mouse monoclonal anti-IK antibody for 1 hour at room temperature. Cells were washed with PBS and incubated with green-fluorescent Alexa Fluor 488 dye-labeled secondary antibody Alexa Fluor 488 goat anti-mouse IgG (Cat #: A11001, Invitrogen, Carlsbad, CA) for 1 hour. Cells were then washed with PBS and counterstained with the DNA-specific nuclear dye DAPI. The coverslips were inverted, mounted onto slides in Vectashield (Vector Labs, Burlinghame, CA) to prevent photobleaching, and sealed with nail varnish. UltraCruz Mounting Medium containing 1.5 μg/ml of 4',6-diamidino-2-phenylindole (DAPI) was purchased from Santa Cruz Biotechnology, Inc.

(Santa Cruz, CA).

References

1. Uckun FM, Ma H, Zhang J, Ozer Z, Dovat S et al. (2012) Serine phosphorylation by SYK is critical for nuclear localization and transcription factor function of Ikaros. Proc. Natl. Acad. Sci. USA 109(44):18072-7. doi: 10.1073/pnas.1209828109

2. Uckun FM, Qazi S, Cely I, Sahin K, Shahidzadeh A et al. (2013) Nanoscale liposomal formulation of a SYK P-site inhibitor against B-precursor leukemia. Blood 121(21):4348-54. doi: 10.1182/blood-2012-11-470633

3. Zhang J, Jackson AF, Naito T, Dose M, Seavitt J, et al. (2011) Harnessing of the nucleosome-remodeling-deacetylase complex controls lymphocyte development and prevents leukemogenesis . Nat Immunol. 13(1): 86-94, 2011

4. Robinson JT, Thorvaldsdóttir H, Winckler W, Guttman M, Lander ES et al. (2011) Integrative Genomics Viewer. Nature Biotechnology 29, 24–26

5. Reich M, Liefeld T, Gould J, Lerner J, Tamayo P et al. (2006) GenePattern 2.0. Nature Genetics 38:500-1

6. Bhojwani D, Kang H, Moskowitz NP, Min DJ, Lee H, Potter JW, et al. (2006) Biologic pathways associated with relapse in childhood acute lymphoblastic leukemia: a Children's Oncology Group study. Blood 108:711-7.

7. Staal FJ, de Ridder D, Szczepanski T, Schonewille T, van der Linden EC et al. (2010) Genome-wide expression analysis of paired diagnosis-relapse samples in ALL indicates involvement of pathways related to DNA replication, cell cycle and DNA repair, independent of immune phenotype. Leukemia 24:491-9.

8. Haferlach T, Kohlmann A, Wieczorek L, Basso G, Kronnie GT et al. (2010) Clinical utility of micro-array based gene expression profiling in the diagnosis and subclassification of leukemia:

report from the International Microarray innovations in leukemia Study Group. J. Clin. Oncol. 28: 2529-2537

9. Liu, WM, Li R, Sun JZ, Wang J, Tsai J et al. (2006) PQN and DQN: Algorithms for expression microarrays, J. Theoretical Biol., 243: 273-278.

10. Mullighan CG, Su X, Zhang J, Radtke I, Phillips LA et al. (2009) Deletion of IKZF1 and Prognois in Aute Lymphoblastic Leukemia. N Engl J Med 360: 470-480

11. Kent WJ. (2002) BLAT--the BLAST-like alignment tool. Genome Res. 12(4):656-64.

12. H. Bengtsson, K. Simpson, J. Bullard & K. Hansen. (2008). Aroma.affymetrix: A generic framework in R for analyzing small to very large Affymetrix data sets in bounded memory, Tech Report #745, Department of Statistics, University of California, Berkeley, February 2008.

13. Bolstad BM, Irizarry RA, Astrand M, Speed TP (2003). A Comparison of Normalization Methods for High Density Oligonucleotide Array Data Based on Bias and Variance. *Bioinformatics* 19(2):185-193

14. Irizarry RA, Bolstad BM, Collin F, Cope LM, Hobbs B et al. (2003) Summaries of Affymetrix GeneChip probe level data *Nucleic Acids Research* 31(4):e15

15. Irizarry, RA, Hobbs, B, Collin, F, Beazer-Barclay, YD, Antonellis, KJ et al. (2003) Exploration, Normalization, and Summaries of High Density Oligonucleotide Array Probe Level Data. *Biostatistics* .Vol. 4, Number 2: 249-264

16. Iacobucci I, Iraci N, Messina M, Lonetti A, Chiaretti S et al. (2012). IKAROS deletions dictate a unique gene expression signature in patients with adult B-cell acute lymphoblastic leukemia. PLoS One. 2012;7(7):e40934. doi: 10.1371/journal.pone.0040934.

17. Subramanian A, Tamayo P, Mootha VK, Mukherjee S, Ebert BL et al., (2005) Gene set enrichment analysis: A knowledge-based approach for interpreting genome-wide expression profiles. Proc Natl Acad Sci U S A. 102: 15545-15550

18. Ozer Z, Qazi S, ishkhanian R, Hasty P, Ma H et al. (2013) Ku Haploinsufficiency Causes a Lymphoproliferative Disorder of Immature T-cell Precursors Due to Ikaros Malfunction.

International Journal of Molecular Medical Science, Vol. 3, No. 7 doi:

10.5376/ijmms.2013.03.0007, 2013

Supplemental Data

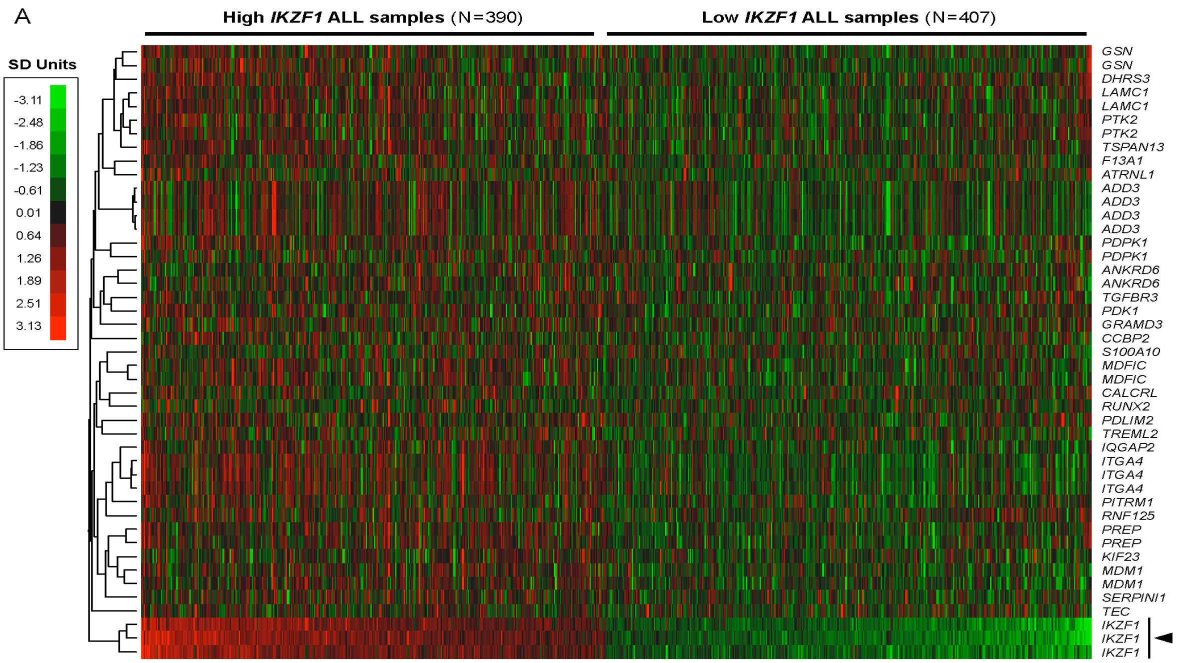

B

Probeset ID	Gene	SD difference (High *IKZF1* (N=390) - Low *IKZF1* (N=407)	T-test P-value	Cluster Order
200696_s_at	*GSN*	0.425	1.5×10^{-9}	1
214040_s_at	*GSN*	0.495	3.6×10^{-12}	2
202481_at	*DHRS3*	0.291	3.5×10^{-5}	3
200770_s_at	*LAMC1*	0.292	7.2×10^{-5}	4
200771_at	*LAMC1*	0.514	1.5×10^{-12}	5
207821_s_at	*PTK2*	0.345	1.5×10^{-6}	6
208820_at	*PTK2*	0.232	8.4×10^{-4}	7
217979_at	*TSPAN13*	0.390	7.1×10^{-9}	8
203305_at	*F13A1*	0.315	4.8×10^{-6}	9
213745_at	*ATRNL1*	0.243	9.5×10^{-4}	10
201034_at	*ADD3*	0.442	5.0×10^{-9}	11
201753_s_at	*ADD3*	0.381	2.3×10^{-7}	12
201752_s_at	*ADD3*	0.489	6.4×10^{-11}	13
205882_x_at	*ADD3*	0.482	1.1×10^{-10}	14
204524_at	*PDPK1*	0.514	4.5×10^{-12}	15
221244_s_at	*PDPK1*	0.238	1.2×10^{-3}	16
204671_s_at	*ANKRD6*	0.162	2.6×10^{-2}	17
204672_s_at	*ANKRD6*	0.191	8.4×10^{-3}	18
204731_at	*TGFBR3*	0.153	3.2×10^{-2}	19
206686_at	*PDK1*	0.366	1.3×10^{-7}	20
218706_s_at	*GRAMD3*	0.216	2.2×10^{-3}	21
206887_at	*CCBP2*	0.194	7.8×10^{-3}	22
200872_at	*S100A10*	0.144	3.8×10^{-2}	23
211675_s_at	*MDFIC*	0.315	5.7×10^{-6}	24
217599_s_at	*MDFIC*	0.418	9.5×10^{-9}	25
210815_s_at	*CALCRL*	0.176	1.3×10^{-2}	26
221282_x_at	*RUNX2*	0.148	3.7×10^{-2}	27
219165_at	*PDLIM2*	0.294	3.4×10^{-5}	28
219748_at	*TREML2*	0.248	7.3×10^{-4}	29
203474_at	*IQGAP2*	0.575	6.5×10^{-16}	30
205884_at	*ITGA4*	0.837	9.4×10^{-30}	31
205885_s_at	*ITGA4*	0.780	5.2×10^{-27}	32
213416_at	*ITGA4*	0.653	3.6×10^{-19}	33
205273_s_at	*PITRM1*	0.474	8.4×10^{-11}	34
207735_at	*RNF125*	0.220	2.8×10^{-3}	35
204117_at	*PREP*	0.534	2.9×10^{-13}	36
37950_at	*PREP*	0.549	2.0×10^{-14}	37
204709_s_at	*KIF23*	0.241	6.5×10^{-4}	38
213761_at	*MDM1*	0.513	6.2×10^{-13}	39
220397_at	*MDM1*	0.540	7.3×10^{-15}	40
205352_at	*SERPINI1*	0.419	7.5×10^{-9}	41
206301_at	*TEC*	0.163	2.1×10^{-2}	42
205038_at	*IKZF1*	2.033	2.9×10^{-178}	43
205039_s_at	*IKZF1*	2.139	8.5×10^{-210}	44
216901_s_at	*IKZF1*	1.968	9.2×10^{-173}	45

Figure S1. Correlation Between Transcript Expression Levels of Ikaros Target Genes in Primary Leukemic Cells from Pediatric ALL Patients. Expression values expressed as

Standard Deviation units were compiled for the 7 studies and rank ordered according to the mean expression of three highly correlated transcripts (205038_at, 205039_s_at, 216901_s_at, 227344_at and 227346_at; 3 of these were common in all Affymetrix platforms - 205038_at, 205039_s_at, 216901_s_at). Heat map depicts up and down regulated transcripts ranging from red to green respectively and clustered according to average distance metric (**A**). T-tests were performed for the combined Standard Deviation units from the 5 datasets (2-sample, Unequal variance correction, P-values<0.05 deemed significant) to reveal 42 transcripts representing 29 genes as significantly up-regulated in samples with high *Ikaros/IKZF1* expression (**B**).

A

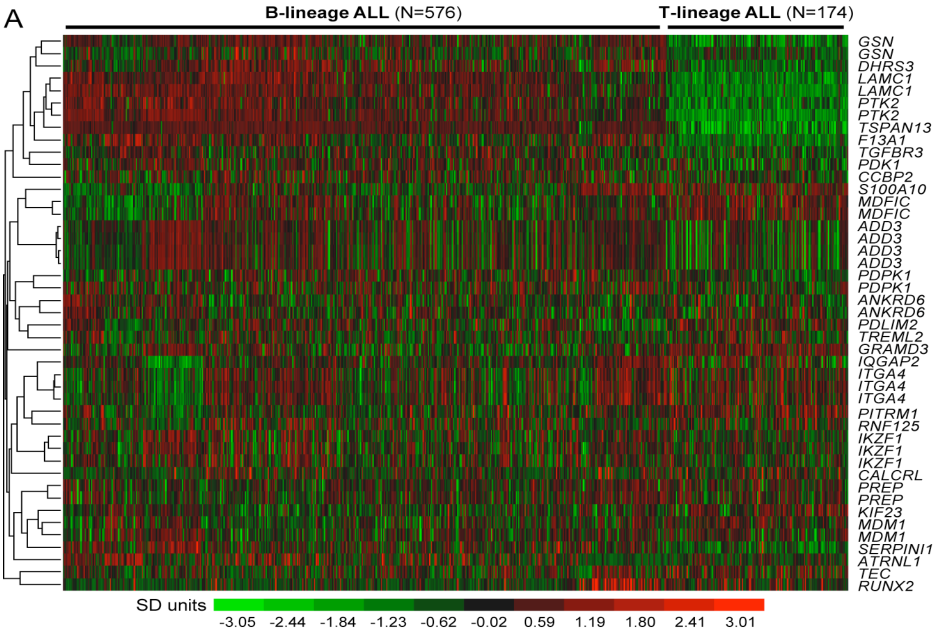

B

Probeset ID	Gene	SD units (B-lineage ALL / T-lineage ALL)	T-Test P-value	Cluster Order
200696_s_at	*GSN*	1.38	1.3×10^{-42}	1
214040_s_at	*GSN*	0.44	5.6×10^{-10}	2
202481_at	*DHRS3*	1.05	2.9×10^{-36}	3
200770_s_at	*LAMC1*	1.62	2.1×10^{-68}	4
200771_at	*LAMC1*	1.72	1.1×10^{-92}	5
207821_s_at	*PTK2*	1.41	1.9×10^{-55}	6
208820_at	*PTK2*	1.90	3.5×10^{-103}	7
217979_at	*TSPAN13*	1.88	8.6×10^{-56}	8
203305_at	*F13A1*	1.18	3.9×10^{-49}	9
204731_at	*TGFBR3*	0.64	3.3×10^{-13}	10
206686_at	*PDK1*	0.63	2.3×10^{-10}	11
206887_at	*CCBP2*	0.35	2.1×10^{-5}	12
200872_at	*S100A10*	-0.53	3.0×10^{-9}	13
211675_s_at	*MDFIC*	-0.71	6.8×10^{-16}	14
217599_s_at	*MDFIC*	-0.60	1.3×10^{-11}	15
201034_at	*ADD3*	0.52	1.0×10^{-5}	16
201753_s_at	*ADD3*	0.51	1.2×10^{-5}	17
201752_s_at	*ADD3*	0.52	3.0×10^{-6}	18
205882_x_at	*ADD3*	0.53	1.2×10^{-6}	19
204524_at	*PDPK1*	0.40	3.7×10^{-7}	20
221244_s_at	*PDPK1*	0.28	1.1×10^{-3}	21
204671_s_at	*ANKRD6*	0.14	1.3×10^{-1}	22
204672_s_at	*ANKRD6*	-0.07	4.7×10^{-1}	23
219165_at	*PDLIM2*	-0.32	1.5×10^{-4}	24
219748_at	*TREML2*	-0.14	9.0×10^{-2}	25
218706_s_at	*GRAMD3*	-0.68	3.3×10^{-18}	26
203474_at	*IQGAP2*	-0.07	5.0×10^{-1}	27
205884_at	*ITGA4*	-0.13	1.3×10^{-1}	28
213416_at	*ITGA4*	-0.32	1.7×10^{-4}	29
205885_s_at	*ITGA4*	-0.24	9.0×10^{-3}	30
205273_s_at	*PITRM1*	-0.57	7.8×10^{-9}	31
207735_at	*RNF125*	-0.07	4.3×10^{-1}	32
205038_at	*IKZF1*	0.23	3.4×10^{-3}	33
205039_s_at	*IKZF1*	0.10	2.2×10^{-1}	34
216901_s_at	*IKZF1*	0.26	1.9×10^{-3}	35
210815_s_at	*CALCRL*	-0.22	1.6×10^{-2}	36
204117_at	*PREP*	-0.08	3.3×10^{-1}	37
37950_at	*PREP*	0.00	9.8×10^{-1}	38
204709_s_at	*KIF23*	-0.43	4.1×10^{-6}	39
213761_at	*MDM1*	-0.37	2.8×10^{-6}	40
220397_at	*MDM1*	-0.05	6.1×10^{-1}	41
205352_at	*SERPINI1*	0.84	8.7×10^{-22}	42
213745_at	*ATRNL1*	0.26	4.4×10^{-4}	43
206301_at	*TEC*	-0.36	2.3×10^{-4}	44
221282_x_at	*RUNX2*	-0.24	5.4×10^{-3}	45

Figure S2. Ikaros Target Gene Transcript Levels in Primary Leukemic Cells from Diagnostic Bone Marrow Specimens of Newly Diagnosed Pediatric ALL Patients. We compiled the archived "The Microarray Innovations in Leukemia" (MILE) study gene expression profiling (GEP) data on primary leukemic cells (GSE13159) from 576 B-lineage ALL/BPL and

174 T-lineage ALL patients. Heat map depicts up and down regulated transcripts ranging from red to green respectively and clustered according to average distance metric (**A**). A cluster of 8 IK target genes (*GSN, DHRS3, LAMC1, PTK2, TSPAN13, F13A1, TGFBR3, PDK1*) exhibited highly significant down regulation in T-lineage ALL. Differential expression levels of *IKZF1* gene (3 transcripts) and 29 IK target genes (42 transcripts) were compared in T-tests utilizing the DQN3 values (2-sample, Unequal variance correction, P-values<0.05 deemed significant). Gene expression values were transformed into standard deviation units and effect sizes were reported using differences standard deviation units between B-lineage ALL and T-lineage ALL (**B**). There was a significant increase in the multi-variate mean of the standard deviation scores for these 45 transcripts comparing B-lineage ALL with T-lineage ALL (MANOVA, $F_{1,748} = 168.7$, $P<0.0001$).

A

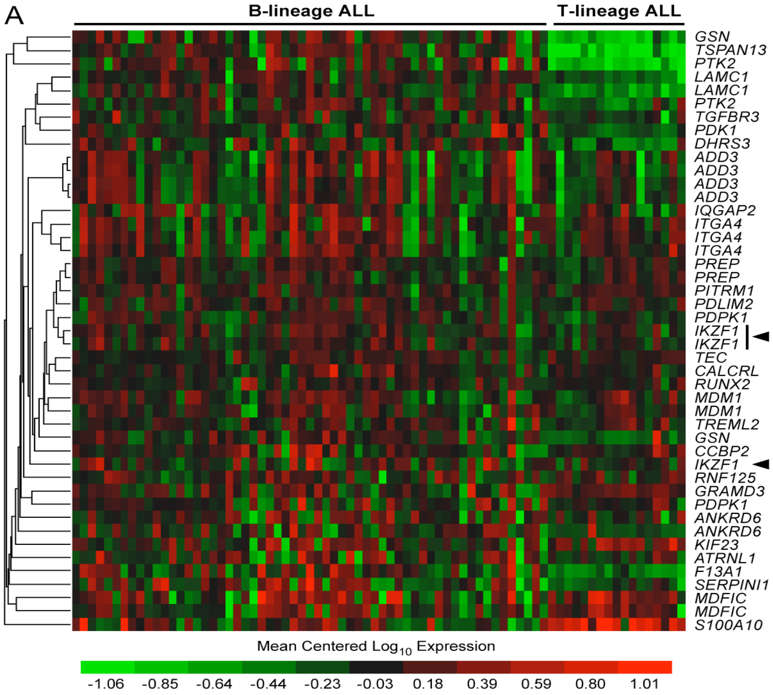

B

Probeset ID	Gene	Fold Increase In B-cell ALL (B/T)	T-Test P-value	Cluster Order
200696_s_at	*GSN*	5.53	2.2 x10^{-11}	1
217979_at	*TSPAN13*	12.90	6.2 x10^{-20}	2
208820_at	*PTK2*	8.43	6.3 x10^{-13}	3
200770_s_at	*LAMC1*	2.19	1.9 x10^{-16}	4
200771_at	*LAMC1*	3.58	4.5 x10^{-13}	5
207821_s_at	*PTK2*	1.81	1.3 x10^{-3}	6
204731_at	*TGFBR3*	1.46	3.5 x10^{-2}	7
206686_at	*PDK1*	1.79	3.5 x10^{-4}	8
202481_at	*DHRS3*	2.40	2.1 x10^{-5}	9
201034_at	*ADD3*	1.39	2.1 x10^{-1}	10
201753_s_at	*ADD3*	1.56	7.4 x10^{-2}	11
201752_s_at	*ADD3*	1.49	5.6 x10^{-2}	12
205882_x_at	*ADD3*	1.52	4.3 x10^{-2}	13
203474_at	*IQGAP2*	1.30	2.7 x10^{-1}	14
205884_at	*ITGA4*	0.89	6.3 x10^{-1}	15
205885_s_at	*ITGA4*	0.90	6.3 x10^{-1}	16
213416_at	*ITGA4*	0.93	7.4 x10^{-1}	17
204117_at	*PREP*	1.06	6.4 x10^{-1}	18
37950_at	*PREP*	0.98	8.9 x10^{-1}	19
205273_s_at	*PITRM1*	0.88	2.8 x10^{-1}	20
219165_at	*PDLIM2*	1.00	9.8 x10^{-1}	21
204524_at	*PDPK1*	1.38	1.5 x10^{-2}	22
205038_at	*IKZF1*	1.15	3.3 x10^{-1}	23
205039_s_at	*IKZF1*	1.19	1.8 x10^{-1}	24
206301_at	*TEC*	0.94	5.1 x10^{-1}	25
210815_s_at	*CALCRL*	1.02	9.0 x10^{-1}	26
221282_x_at	*RUNX2*	1.03	7.9 x10^{-1}	27
213761_at	*MDM1*	0.93	7.1 x10^{-1}	28
220397_at	*MDM1*	1.03	8.6 x10^{-1}	29
219748_at	*TREML2*	0.96	8.2 x10^{-1}	30
214040_s_at	*GSN*	1.74	1.4 x10^{-3}	31
206887_at	*CCBP2*	0.88	5.3 x10^{-1}	32
216901_s_at	*IKZF1*	1.10	6.2 x10^{-1}	33
207735_at	*RNF125*	0.96	8.1 x10^{-1}	34
218706_s_at	*GRAMD3*	0.64	2.4 x10^{-2}	35
221244_s_at	*PDPK1*	1.02	9.3 x10^{-1}	36
204672_s_at	*ANKRD6*	1.09	7.0 x10^{-1}	37
204671_s_at	*ANKRD6*	1.47	6.6 x10^{-2}	38
204709_s_at	*KIF23*	0.51	3.4 x10^{-3}	39
213745_at	*ATRNL1*	1.13	6.3 x10^{-1}	40
203305_at	*F13A1*	2.89	1.6 x10^{-5}	41
205352_at	*SERPINI1*	1.69	5.4 x10^{-2}	42
211675_s_at	*MDFIC*	0.51	5.7 x10^{-2}	43
217599_s_at	*MDFIC*	0.68	1.2 x10^{-1}	44
200872_at	*S100A10*	0.20	3.6 x10^{-8}	45

Figure S3. Ikaros Target Gene Transcript Levels in Primary Leukemic Cells from Diagnostic Bone Marrow Specimens of Newly Diagnosed Pediatric ALL Patients who

Subsequently Experienced a Relapse. Expression levels of IK target genes were also examined for primary leukemic cells from matched-pair diagnosis vs. relapse specimens of B-lineage ALL/BPL patients using archived GEP datasets from 2 independent studies (GSE 3912, GSE18497). Gene expression values for leukemia cells obtained from 59 B-lineage ALL patients at diagnosis (GSE3912, N=32 and GSE18497, N=27) were compared to the gene expression values for leukemia cells obtained from 17 T-lineage ALL patients at diagnosis (GSE3912, N=3 and GSE18497, N=14). RMA-normalized values for the GSE18497 dataset and the MAS5- Signal intensity values for the GSE3912 dataset were log_{10} transformed and mean-centered to the average value for the diagnosis samples for each gene transcript in each study. Heat map depicts up and down regulated transcripts ranging from red to green respectively for expression values and clustered according to average distance metric (**A**). T-tests (2 sample) compared B- versus T-lineage ALL subsets for IK target gene expression levels in leukemic cells from initial diagnosis specimens of patients who subsequently experienced a relapse (**B**). There was a significant increase in the multi-variate mean for the 45 transcripts representing *IKZF1* gene (3 transcripts) and 29 IK target genes (42 transcripts) in B-lineage ALL compared with T-lineage ALL (MANOVA, $F_{1,74} = 9.32$, $P=0.0032$).

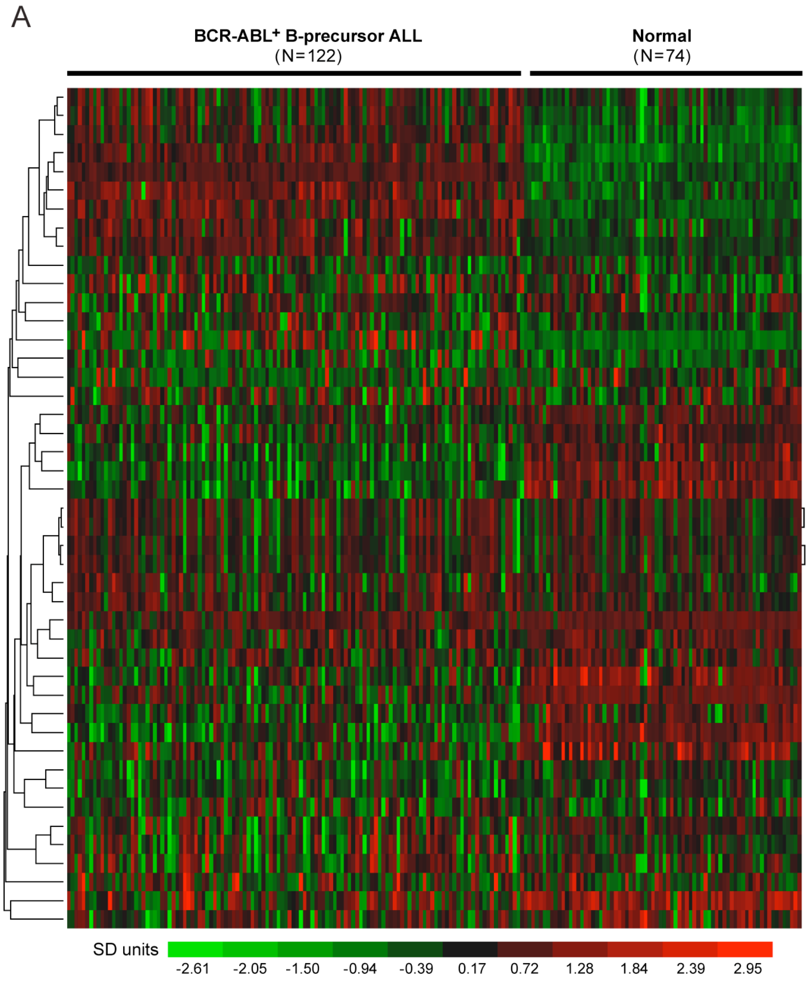

B

Probeset ID	Gene	SD units (B-lineage ALL - Normal)	T-test P-value	Cluster Order
205884_at	*ITGA4*	0.53	1.6×10^{-6}	1
205885_s_at	*ITGA4*	0.82	1.1×10^{-12}	2
213416_at	*ITGA4*	1.12	1.9×10^{-22}	3
200771_at	*LAMC1*	1.34	3.8×10^{-41}	4
217979_at	*TSPAN13*	1.13	3.9×10^{-38}	5
200770_s_at	*LAMC1*	1.23	3.8×10^{-26}	6
202481_at	*DHRS3*	1.27	3.6×10^{-40}	7
207821_s_at	*PTK2*	0.80	1.3×10^{-12}	8
208820_at	*PTK2*	0.74	1.2×10^{-22}	9
205273_s_at	*PITRM1*	0.07	5.3×10^{-1}	10
207735_at	*RNF125*	0.62	2.4×10^{-5}	11
206686_at	*PDK1*	-0.17	2.0×10^{-1}	12
218706_s_at	*GRAMD3*	0.22	5.7×10^{-2}	13
213745_at	*ATRNL1*	1.10	1.3×10^{-16}	14
206301_at	*TEC*	0.18	1.3×10^{-1}	15
221282_x_at	*RUNX2*	-0.36	6.3×10^{-3}	16
206887_at	*CCBP2*	0.15	3.1×10^{-1}	17
203474_at	*IQGAP2*	-0.56	5.1×10^{-8}	18
205352_at	*SERPINI1*	-0.58	6.1×10^{-9}	19
220397_at	*MDM1*	-1.04	2.7×10^{-14}	20
213761_at	*MDM1*	-1.41	2.9×10^{-33}	21
204709_s_at	*KIF23*	-1.36	6.7×10^{-21}	22
201752_s_at	*ADD3*	-0.01	9.0×10^{-1}	23
205882_x_at	*ADD3*	0.04	6.9×10^{-1}	24
201034_at	*ADD3*	0.00	9.9×10^{-1}	25
201753_s_at	*ADD3*	0.02	8.5×10^{-1}	26
217599_s_at	*MDFIC*	0.21	6.1×10^{-2}	27
211675_s_at	*MDFIC*	0.12	1.8×10^{-1}	28
200696_s_at	*GSN*	-0.24	9.1×10^{-5}	29
203305_at	*F13A1*	-0.32	9.6×10^{-4}	30
219165_at	*PDLIM2*	-0.28	1.5×10^{-2}	31
204731_at	*TGFBR3*	-1.12	8.2×10^{-15}	32
200872_at	*S100A10*	-0.88	1.1×10^{-19}	33
204672_s_at	*ANKRD6*	-0.71	1.1×10^{-7}	34
204671_s_at	*ANKRD6*	-1.32	8.5×10^{-27}	35
219748_at	*TREML2*	-0.96	9.4×10^{-10}	36
37950_at	*PREP*	-0.32	7.3×10^{-3}	37
204117_at	*PREP*	-0.03	8.3×10^{-1}	38
221244_s_at	*PDPK1*	0.04	7.6×10^{-1}	39
205038_at	*IKZF1*	-0.34	1.2×10^{-2}	40
205039_s_at	*IKZF1* ◄	0.12	4.0×10^{-1}	41
216901_s_at	*IKZF1*	-0.01	9.5×10^{-1}	42
210815_s_at	*CALCRL*	0.11	4.7×10^{-1}	43
214040_s_at	*GSN*	-0.89	1.0×10^{-9}	44
204524_at	*PDPK1*	-0.51	1.2×10^{-3}	45

Figure S4. Ikaros Target Gene Transcript Levels in Primary Leukemia Cells from BCR-ABL+ B-precursor ALL (BPL) Patients Compared to Normal Bone Marrow Cells. We compiled the archived "Microarray Innovations in Leukemia" (MILE) study gene expression profiling (GEP) data on primary leukemic cells (GSE13159) from 122 pediatric BCR-ABL+ B-precursor ALL (BPL) patients with t(9;22) translocation and normal hematopoietic cells from 74 non-leukemic bone marrow specimens. Differential expression levels of *IKZF1* gene (3 transcripts) and 29 IK target genes (42 transcripts) were compared in T-tests utilizing the DQN3 values (2-sample, Unequal variance correction, p-values<0.05 deemed significant). Gene expression values were transformed into standard deviation units calculated from the mean and

standard deviation expression values for all the samples in each study and effect sizes were reported using differences standard deviation units between comparison groups. Heat map depicts up and down regulated transcripts ranging from red to green respectively for expression values and clustered according to average distance metric (**A**). Statistical differences were assessed utilizing MANOVA that showed no effect in the multivariate mean for the 45 transcripts in BCR-ABL$^+$ BPL cells ($F_{1,94}$ = 0.768, P = 0.382) and differences in the individual transcripts were reported using T-tests assuming unequal variances (**B**). Thirteen (7 genes) and 18 transcripts (15 genes) were up regulated and down regulated respectively (P<0.05).

A

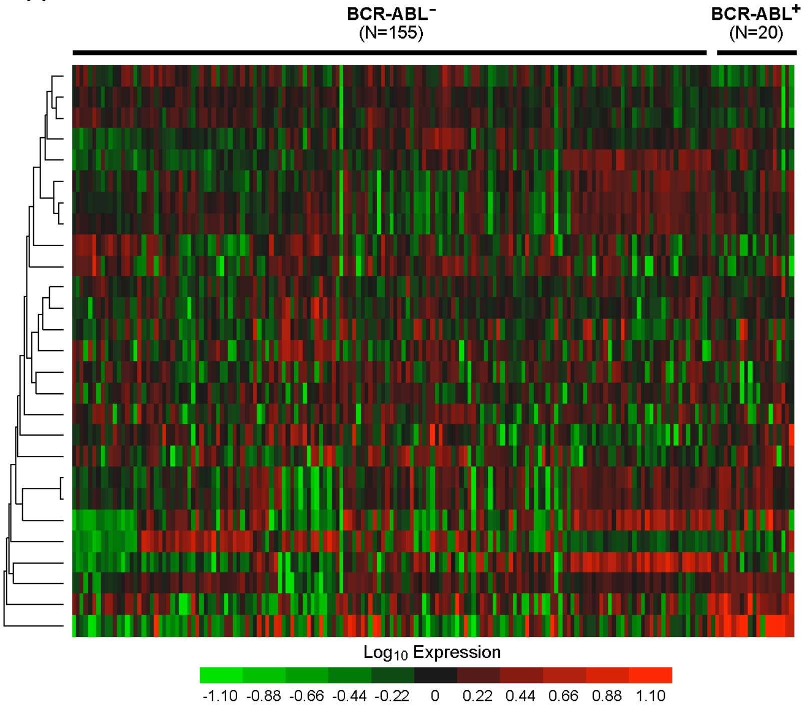

B

Probeset ID	Gene	Fold Change (Increase in BCR-ABL$^+$)	T-Test P-value	Cluster Order
201416_at	*SOX4*	0.60	1.0×10^{-2}	1
201417_at	*SOX4*	0.79	3.0×10^{-1}	2
201418_s_at	*SOX4*	0.66	3.1×10^{-2}	3
203408_s_at	*SATB1*	1.26	2.1×10^{-2}	4
218902_at	*NOTCH1*	0.97	8.5×10^{-1}	5
207968_s_at	*MEF2C*	1.17	2.5×10^{-1}	6
209199_s_at	*MEF2C*	1.16	1.9×10^{-1}	7
209200_at	*MEF2C*	1.24	6.3×10^{-2}	8
205798_at	*IL7R*	0.58	6.3×10^{-3}	9
213668_s_at	*SOX4*	0.72	1.5×10^{-1}	10
207445_s_at	*CCR9*	1.04	7.3×10^{-1}	11
208025_s_at	*HMGA2*	1.08	3.6×10^{-1}	12
221282_x_at	*RUNX2*	1.20	3.5×10^{-1}	13
216994_s_at	*RUNX2*	1.26	1.5×10^{-1}	14
214447_at	*ETS1*	0.71	1.3×10^{-1}	15
221283_at	*RUNX2*	0.86	4.7×10^{-1}	16
213665_at	*SOX4*	0.91	6.8×10^{-1}	17
203104_at	*CSF1R*	1.49	1.9×10^{-2}	18
206631_at	*PTGER2*	1.34	1.8×10^{-1}	19
204661_at	*CD52*	1.65	4.6×10^{-4}	20
34210_at	*CD52*	1.61	3.1×10^{-4}	21
207339_s_at	*LTB*	1.56	1.2×10^{-1}	22
206674_at	*FLT3*	0.70	1.3×10^{-1}	23
206591_at	*RAG1*	0.83	4.0×10^{-1}	24
210487_at	*DNTT*	1.84	9.4×10^{-6}	25
201445_at	*CNN3*	3.57	3.0×10^{-7}	26
212592_at	*IGJ*	7.46	1.8×10^{-6}	27

Figure S5. Lymphoid Priming Gene Transcript Levels in Primary Leukemia Cells from Pediatric BCR-ABL$^-$ and BCR-ABL$^+$ BPL Patients. Expression levels of lymphoid priming gene transcripts were compared for primary leukemic cells from 155 pediatric BCR-ABL$^-$ BPL patients vs. 20 BCR-ABL$^+$ BPL patients on the Mullighan study (GSE12995). Transcript signal values were obtained from hybridization onto the Affymetrix Human Genome U133A gene chip arrays. Heat map depicts up and down regulated transcripts ranging from red to green respectively for mean centered $\log_{10}$ transformed expression values and clustered according to average distance metric.

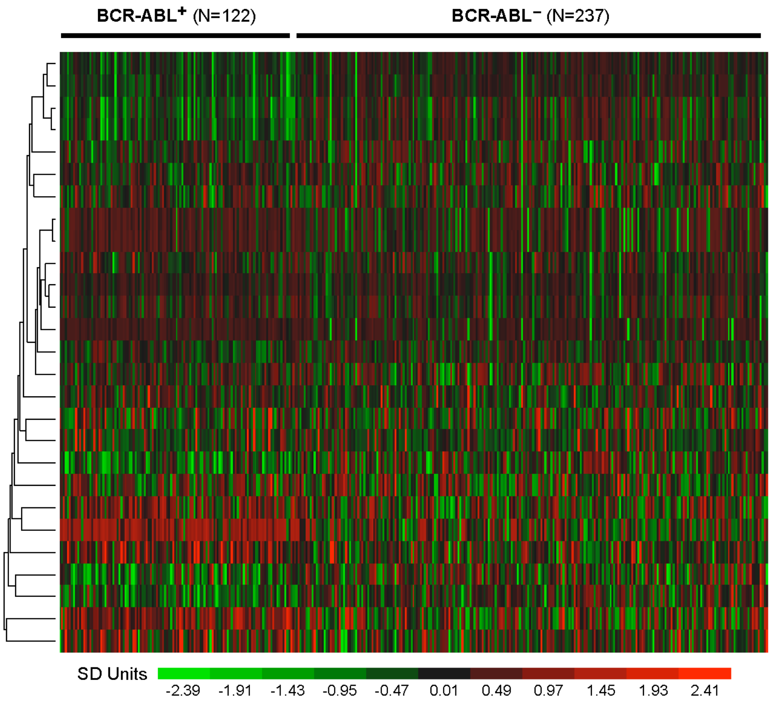

Probeset ID	Gene	SD units (Increase in BCR-ABL+)	T-Test P-value	Cluster Order
201416_at	*SOX4*	-0.33	1.9×10^{-7}	1
201417_at	*SOX4*	-0.38	1.6×10^{-8}	2
201418_s_at	*SOX4*	-0.53	1.8×10^{-10}	3
213668_s_at	*SOX4*	-0.47	2.0×10^{-9}	4
206674_at	*FLT3*	-0.08	2.5×10^{-1}	5
203408_s_at	*SATB1*	-0.04	5.6×10^{-1}	6
218902_at	*NOTCH1*	0.01	8.7×10^{-1}	7
204661_at	*CD52*	0.28	3.1×10^{-6}	8
34210_at	*CD52*	0.30	7.3×10^{-7}	9
207968_s_at	*MEF2C*	0.10	1.6×10^{-1}	10
209199_s_at	*MEF2C*	0.15	3.4×10^{-4}	11
209200_at	*MEF2C*	0.13	2.7×10^{-2}	12
210487_at	*DNTT*	0.26	1.3×10^{-6}	13
207445_s_at	*CCR9*	-0.06	2.5×10^{-1}	14
206591_at	*RAG1*	0.06	4.6×10^{-1}	15
208025_s_at	*HMGA2*	0.12	1.3×10^{-1}	16
216994_s_at	*RUNX2*	-0.01	9.2×10^{-1}	17
221282_x_at	*RUNX2*	0.06	5.5×10^{-1}	18
213665_at	*SOX4*	-0.63	1.2×10^{-9}	19
221283_at	*RUNX2*	0.08	4.6×10^{-1}	20
207339_s_at	*LTB*	0.40	1.1×10^{-4}	21
212592_at	*IGJ*	0.96	8.9×10^{-27}	22
203104_at	*CSF1R*	0.53	9.2×10^{-7}	23
205798_at	*IL7R*	-0.56	4.1×10^{-6}	24
214447_at	*ETS1*	-0.53	1.0×10^{-7}	25
201445_at	*CNN3*	0.64	5.6×10^{-10}	26
206631_at	*PTGER2*	0.41	5.6×10^{-4}	27

Figure S6. Lymphoid Priming Gene Transcript Levels in Primary Leukemia Cells from BCR-ABL$^-$ and BCR-ABL$^+$ Pediatric BPL Patients from the MILE Study. Expression levels of lymphoid priming transcripts were compared in primary leukemic cells from BCR-ABL$^-$ B-precursor ALL patients *vs.* BCR-ABL$^+$ B-precursor ALL patients on MILE study (GSE13159). Transcript signal values were obtained from hybridization onto the Affymetrix Human Genome U133A genechip arrays. Heat map depicts up and down regulated transcripts ranging from red to green respectively for standardized expression values and clustered according to average distance metric. Multivariate means between these two groups was not significant (MANOVA, $F_{1,357} = 1.664$, $P = 0.198$).

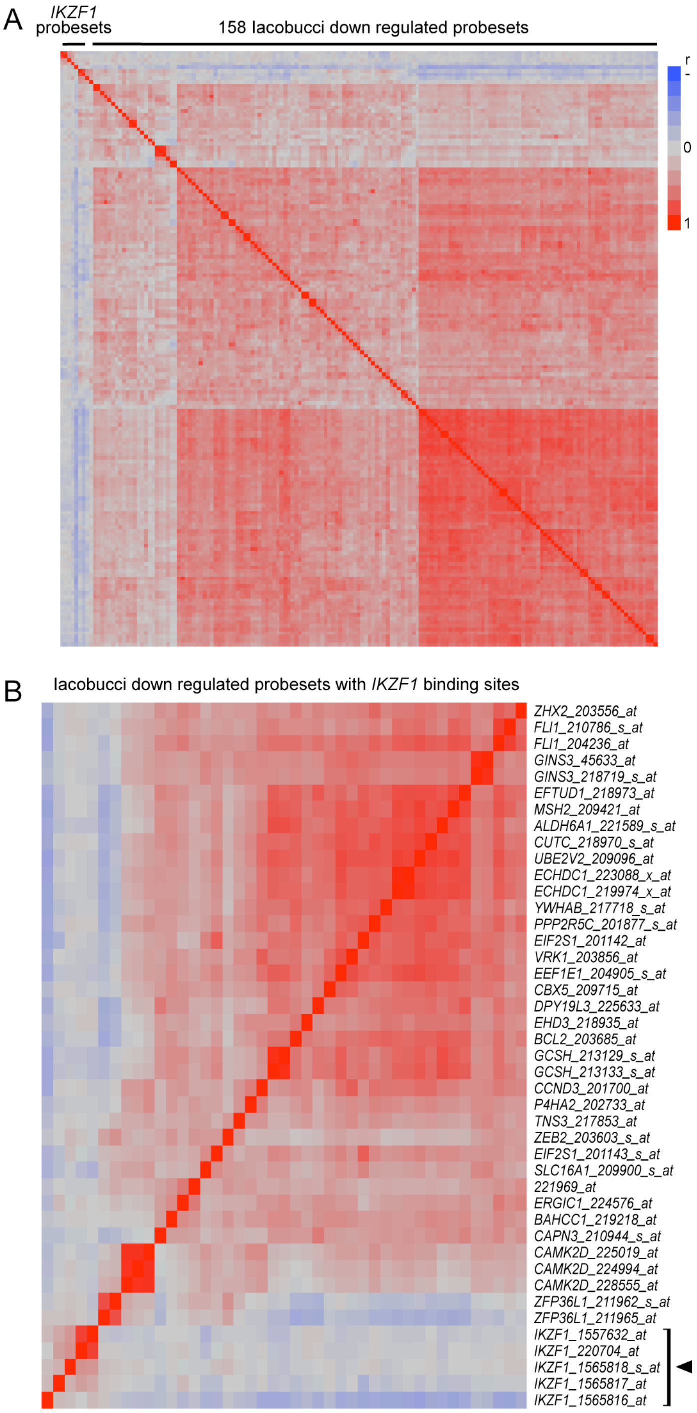

Figure S7. Correlation Cluster of Gene Expression Profiles of Primary Leukemic Cells from Pediatric BPL Patients Forms Distinct Modules of Expression for *IKZF1* vs. Iacobucci Signature Genes for Adult Ph$^+$ BPL with *IKZF1* Deletions [A] Pairwise correlations were performed using the adult gene signature for *IKZF1* deletions (158 probe sets

down regulated in adult Ph^+ BPL patients with *IKZF1* deletions [16] and the 5 *IKZF1* probe sets for Exons 1-4 for pediatric BPL cases using the RMA normalized dataset (N=123 Ph^+ cases and 327 Ph^- cases). Correlation coefficients (r) were determined between all gene pairs and cluster analysis was applied to the matrix of correlation coefficients for both rows and columns of probe set identifications whereby red indicated positive correlations between probe set pairs and blue indicated negative correlation between probe set pairs. The majority of correlations between the 158 probe sets were significantly positive in direction (92% with P<0.05 (11456/12403 pairs) (Median correlation coefficient = 0.43; Interquartile Range = 0.28 to 0.57), whereas the majority of correlations for each of the *IKZF1* probe sets with each of the 158 probe sets for the adult *IKZF1* deletion signature exhibited either a positive correlation (42% with P<0.05 [329/790 pairs]) or no correlation (54% with P>0.05 [428/790 pairs], Median correlation coefficient = -0.07, Interquartile Range = -0.16 to -0.01) in pediatric BPL cases. [B] Probeset correlations were clustered for a subset of 29 IK target genes that harbored IK binding sites in mice identified by cross-referencing this gene set with the archived CHiPseq data (GSM803110 archived in GSE32311 [3] and *IKZF1* probe sets specific for Exons 1-4 in pediatric BPL samples.

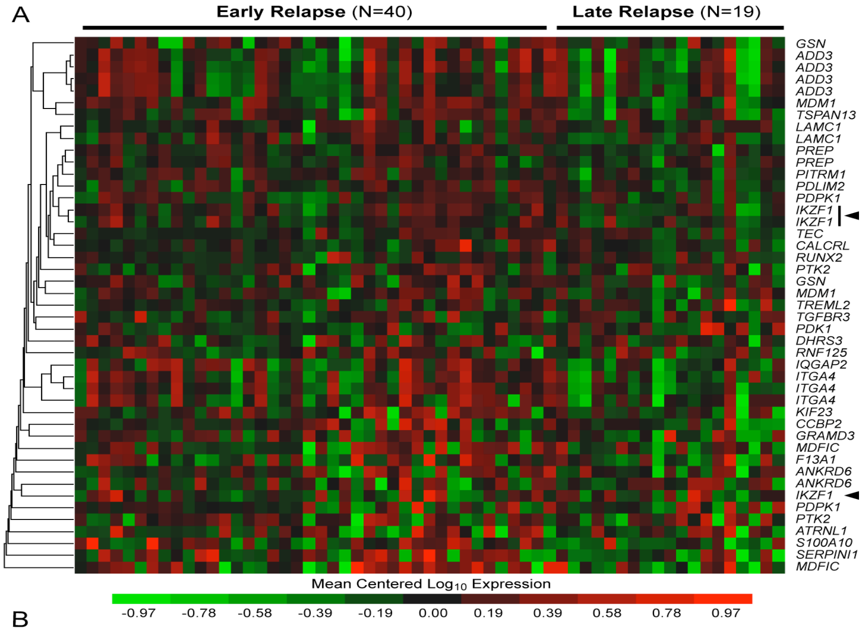

B

Probeset ID	Gene Symbol	Fold Change (Early/Late at Diag)	T-test P-value	Cluster Order
200696_s_at	*GSN*	1.26	0.3346	1
201034_at	*ADD3*	1.74	0.0301	2
201753_s_at	*ADD3*	1.55	0.0851	3
201752_s_at	*ADD3*	1.40	0.1195	4
205882_x_at	*ADD3*	1.32	0.1888	5
213761_at	*MDM1*	1.88	0.0003	6
217979_at	*TSPAN13*	1.59	0.0198	7
200770_s_at	*LAMC1*	0.98	0.8731	8
200771_at	*LAMC1*	1.31	0.0780	9
204117_at	*PREP*	1.37	0.0102	10
37950_at	*PREP*	1.19	0.1641	11
205273_s_at	*PITRM1*	1.35	0.0219	12
219165_at	*PDLIM2*	1.29	0.0587	13
204524_at	*PDPK1*	1.08	0.5813	14
205038_at	*IKZF1*	1.32	0.0633	15
205039_s_at	*IKZF1*	1.14	0.3265	16
206301_at	*TEC*	1.11	0.3146	17
210815_s_at	*CALCRL*	1.17	0.2555	18
221282_x_at	*RUNX2*	1.00	0.9863	19
207821_s_at	*PTK2*	1.05	0.8014	20
214040_s_at	*GSN*	1.21	0.2434	21
220397_at	*MDM1*	1.18	0.3459	22
219748_at	*TREML2*	0.93	0.6815	23
204731_at	*TGFBR3*	1.03	0.8713	24
206686_at	*PDK1*	0.74	0.0740	25
202481_at	*DHRS3*	1.02	0.9165	26
207735_at	*RNF125*	1.03	0.8938	27
203474_at	*IQGAP2*	1.79	0.0110	28
205885_s_at	*ITGA4*	1.68	0.0265	29
213416_at	*ITGA4*	2.07	0.0005	30
205884_at	*ITGA4*	1.69	0.0510	31
204709_s_at	*KIF23*	1.35	0.2028	32
206887_at	*CCBP2*	1.47	0.0733	33
218706_s_at	*GRAMD3*	1.28	0.2502	34
217599_s_at	*MDFIC*	1.34	0.2414	35
203305_at	*F13A1*	1.40	0.1871	36
204672_s_at	*ANKRD6*	1.27	0.3188	37
204671_s_at	*ANKRD6*	0.90	0.6397	38
216901_s_at	*IKZF1*	1.04	0.8673	39
221244_s_at	*PDPK1*	0.98	0.9371	40
208820_at	*PTK2*	0.98	0.9434	41
213745_at	*ATRNL1*	0.99	0.9741	42
200872_at	*S100A10*	1.89	0.0143	43
205352_at	*SERPINI1*	1.74	0.0598	44
211675_s_at	*MDFIC*	1.56	0.2539	45

Figure S8. Ikaros target gene transcript levels in primary leukemia cells from newly diagnosed pediatric BPL patients who subsequently experience an early vs. late relapse. Expression levels of IK target genes were examined for primary leukemic cells from matched-pair diagnosis *vs.* relapse specimens of BPL patients using archived GEP datasets from 2 independent studies (GSE 3912, GSE18497). IK target gene expression levels were compared

for leukemic cells from initial diagnostic specimens of patients who subsequently experienced an early relapse (N=40; <36 months) versus a late relapse (N=19; >36months) (2-sample T-test). Heat map depicts up and down regulated transcripts ranging from red to green respectively for $\log_{10}$ transformed mean centered expression values and clustered according to average distance metric (**A**). T-tests (2 sample) compared for Early versus Late relapse subsets for IK target gene expression levels in leukemic cells from initial diagnosis specimens of these patients. (**B**). There was a significant increase in the multivariate mean for 45 transcripts (3 transcripts for *IKZF*1 and 42 transcripts for 29 IK target genes) comparing Early to Late Relapse subsets (Average fold increase = 1.3; MANOVA, $F_{1,57} = 7.11$, $P=0.01$).

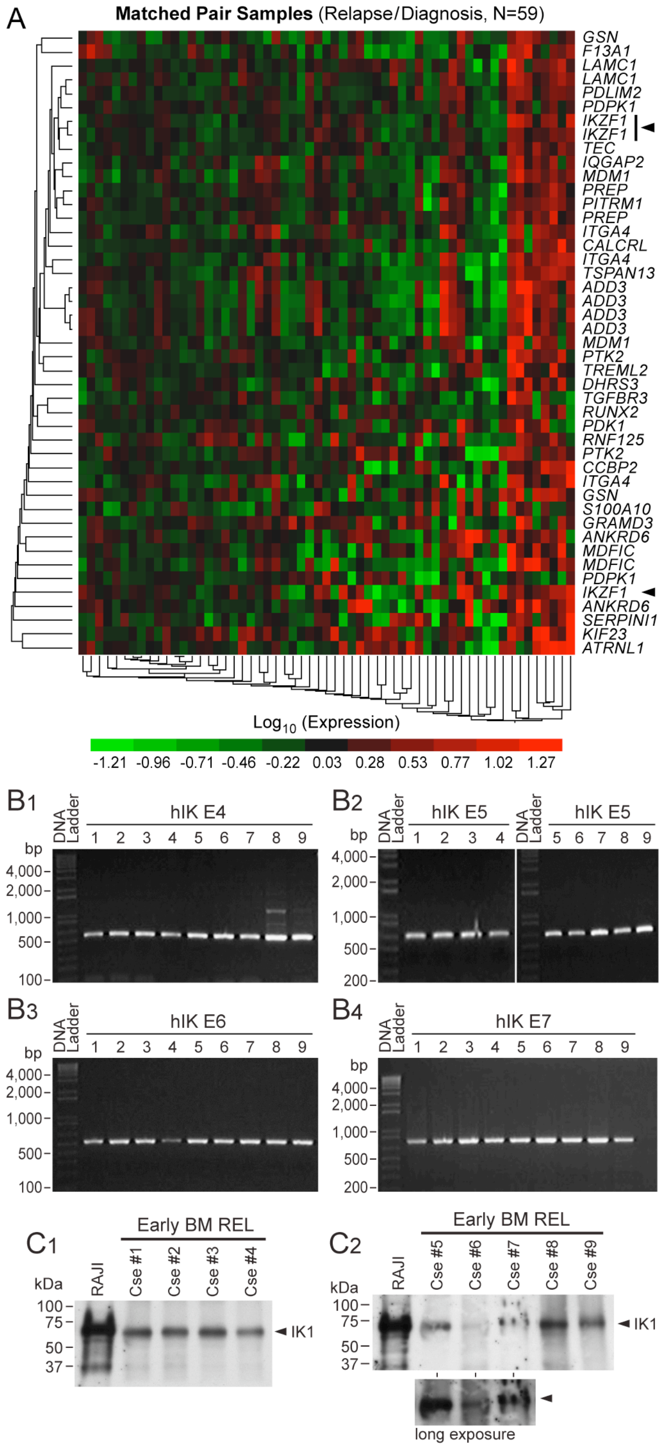

Figure S9. Primary Leukemia Cells from Pediatric BPL Patients in Relapse are not Characterized by Ikaros Deficiency. [A] Matched pair gene expression values for leukemia cells obtained from 59 BPL patients at diagnosis and then at relapse (combined from GSE3912, N=32 and GSE18497, N=27). RMA-normalized values for the GSE18497

dataset and the MAS5- Signal intensity values for the GSE3912 dataset were $\log_{10}$ transformed and mean-centered to the average value for the diagnosis samples for each gene transcript in each study. To determine the differential expression of each gene, paired T-tests were performed for the combined mean-centered values from GSE3910 and GSE18497 datasets (Unequal variance correction, P<0.05 deemed significant). Heat map depicts up and down regulated transcripts ranging from red to green respectively for standardized expression values and clustered according to average distance metric. **[B]** Exon-specific genomic PCR with DNA sequencing on purified genomic DNA samples from 9 patients with BPL in first bone marrow relapse that occurred within 12 months of the completion of primary therapy . *IKZF1* Exons E4-7 and their intron-exon junctions were PCR amplified, as described in Materials and Methods using the PCR primers listed in **Table S3**. Normal size PCR products were obtained in each of the 9 patients, providing strong evidence against the existence of homozygous deletions of the entire *IKZF1* locus or within *IKZF1* exons 4-7. **[C]** IK Western blot analysis on whole cell lysates was performed as described in Materials and Methods. See text for discussion.

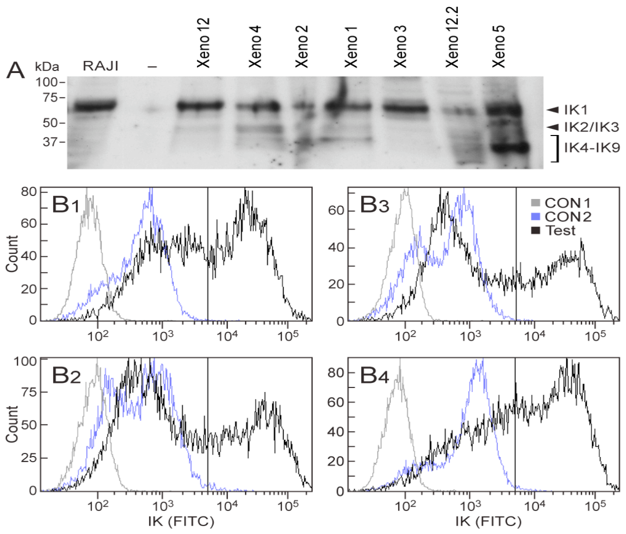

B5

	Mean Channel of Immunofluorescence			
	B1	B2	B3	B4
Unstained	99	142	94	87
2° Ab alone	701	844	728	1253
αIK Ab + 2° Ab	20,124	21,471	20,115	22,117

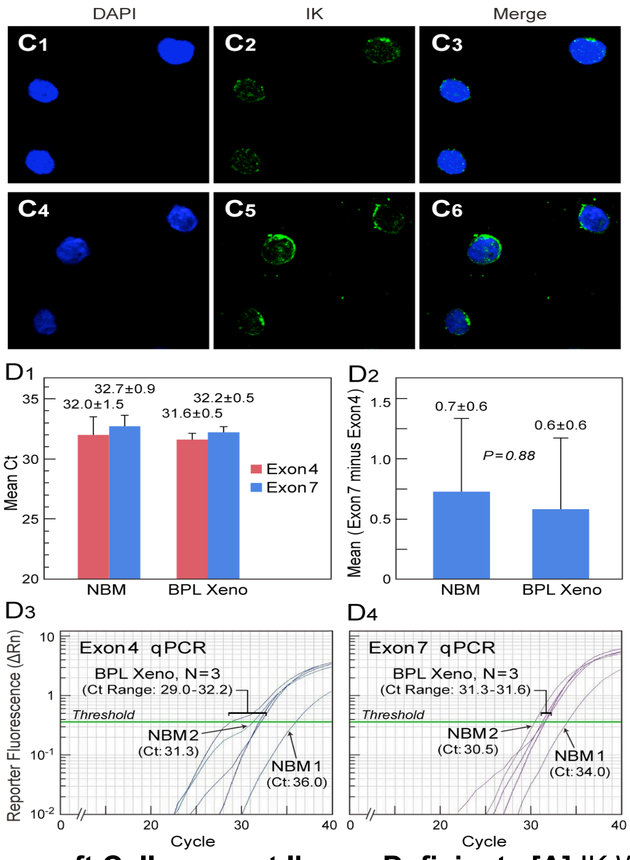

Figure S10. BPL Xenograft Cells are not Ikaros-Deficient. [A] IK Western blot analysis was performed on ALL xenograft cells (and RAJI cell line that was included as a positive control).

Xenograft cells were $CD10^{+}CD19^{+}CD34^{+}HLA\text{-}DR^{+}$ and caused overt leukemia in NOD/SCID mice after reinjection [2]. **[B]** IK expression in BPL xenograft cells was also detected by intracellular flow cytometry. B1 = Xeno-1 in A; B2 = Xeno-2 in A; B3 = Xeno-3 in A; B4 = Xeno-12.2 in A. **[C]** Confocal fluorescence microscopy showed punctate nuclear and perinuclear localization of IK in xenograft cells from two separate cases (C1-C3 = Xeno-1 in A and C4-C6 = Xeno-5 in A). System Magnification: 630 (Magnification[objective]: 63 x Magnification[Eyepiece]: 10). **[D]** Shown in D1 are the mean Ct values for Exon 4 and Exon 7 amplicons obtained by qPCR of genomic DNA samples from 3 BPL xenograft cases (Xeno-1, Xeno-2, and Xeno-5 in A) and normal hematopoietic cells from 2 non-leukemic normal bone marrow specimens in two independent experiments. Depicted in D2 are the bar graphs of Ct data from [D1] for Exon 4-specific qPCR after normalization by referencing the raw data to the Ct values from Exon 7-specific qPCR. Depicted in D3 and D4 the are representative cycle number vs. Log (ΔRn) (fluorescence signal) amplification plots obtained using the *IKZF1* Exon 4-specific test primers and *IKZF1* Exon 7-specific reference primers respectively for the normal hematopoietic cells and the 3 BPL xenograft cases.

Table S1. Expression Levels of Ikaros Target Genes in Ph(BCR-ABL)$^+$ vs. Ph(BCR-ABL)$^-$ BPL Patients – Mullighan Study

Probeset ID	Gene	Fold Increase In BCR-ABL positive cells	T-Test P-value	Cluster Order
205884_at	*ITGA4*	1.50	3.5 x10^{-4}	1
205885_s_at	*ITGA4*	1.45	1.4 x10^{-3}	2
213416_at	*ITGA4*	1.41	7.2 x10^{-4}	3
203474_at	*IQGAP2*	1.27	1.0 x10^{-2}	4
37950_at	*PREP*	0.92	3.6 x10^{-1}	5
204117_at	*PREP*	0.96	4.8 x10^{-1}	6
**205273_s_at**	***PITRM1***	**0.86**	**1.3 x10^{-2}**	**7**
213761_at	*MDM1*	0.88	1.5 x10^{-1}	8
205038_at	*IKZF1*	0.88	5.7 x10^{-1}	9
205039_s_at	*IKZF1*	0.93	6.7 x10^{-1}	10
217979_at	*TSPAN13*	1.56	4.9 x10^{-7}	11
200771_at	*LAMC1*	1.11	1.3 x10^{-1}	12
200770_s_at	*LAMC1*	1.29	6.1 x10^{-3}	13
207735_at	*RNF125*	1.31	3.0 x10^{-2}	14
201752_s_at	*ADD3*	1.11	5.8 x10^{-1}	15
205882_x_at	*ADD3*	1.09	6.4 x10^{-1}	16
201753_s_at	*ADD3*	1.20	4.0 x10^{-1}	17
201034_at	*ADD3*	0.90	5.7 x10^{-1}	18
207821_s_at	*PTK2*	0.84	3.4 x10^{-1}	19
219165_at	*PDLIM2*	1.08	5.2 x10^{-1}	20
206887_at	*CCBP2*	0.83	2.3 x10^{-1}	21
204731_at	*TGFBR3*	0.88	2.5 x10^{-1}	22
206301_at	*TEC*	0.80	1.1 x10^{-1}	23
200696_s_at	*GSN*	1.89	4.3 x10^{-4}	24
204672_s_at	*ANKRD6*	1.05	7.6 x10^{-1}	25
221282_x_at	*RUNX2*	1.20	3.5 x10^{-1}	26
200872_at	*S100A10*	1.32	1.8 x10^{-1}	27
210815_s_at	*CALCRL*	1.22	4.1 x10^{-1}	28
219748_at	*TREML2*	1.83	5.3 x10^{-3}	29
220397_at	*MDM1*	1.07	7.2 x10^{-1}	30
**204709_s_at**	***KIF23***	**0.62**	**5.6 x10^{-2}**	**31**
205352_at	*SERPINI1*	0.94	8.1 x10^{-1}	32
202481_at	*DHRS3*	1.44	1.5 x10^{-1}	33
203305_at	*F13A1*	0.94	7.3 x10^{-1}	34
204524_at	*PDPK1*	0.95	8.1 x10^{-1}	35
221244_s_at	*PDPK1*	1.23	3.7 x10^{-1}	36
217599_s_at	*MDFIC*	2.12	6.4 x10^{-4}	37
211675_s_at	*MDFIC*	2.25	5.7 x10^{-4}	38
204671_s_at	*ANKRD6*	0.55	1.7 x10^{-2}	39
206686_at	*PDK1*	0.90	6.7 x10^{-1}	40
216901_s_at	*IKZF1*	1.24	3.8 x10^{-1}	41
218706_s_at	*GRAMD3*	1.38	2.0 x10^{-1}	42
208820_at	*PTK2*	0.93	7.8 x10^{-1}	43
213745_at	*ATRNL1*	1.14	6.9 x10^{-1}	44
214040_s_at	*GSN*	2.67	1.5 x10^{-3}	45

Expression levels of IK target genes were compared in primary leukemic cells from 155 pediatric BCR-ABL$^-$ BPL patients and 20 BCR-ABL$^+$ BPL patients on the Mullighan study (GSE12995). Trimmed mean target intensity of each array was globally scaled to 500 (MAS5

values) as the normalization method. T-tests were performed using $\log_{10}$ transformed MAS5 signal values (2-sample, Unequal variance correction, P-values<0.05 deemed significant) to identify differentially regulated transcripts for IK target genes.

Table S2. Expression Levels of Ikaros Target Genes in BCR-ABL$^+$ vs. BCR-ABL$^-$ BPL Patients – MILE Study

Probeset ID	Gene	SD units<br>With t(9;22) - Without t(9;22)	T-Test P-value	Cluster Order
200696_s_at	*GSN*	0.49	3.4 x10^{-10}	1
214040_s_at	*GSN*	0.51	1.6 x10^{-5}	2
219165_at	*PDLIM2*	0.25	1.4 x10^{-2}	3
203305_at	*F13A1*	0.42	6.1 x10^{-6}	4
200770_s_at	*LAMC1*	0.40	3.0 x10^{-7}	5
200771_at	*LAMC1*	0.18	7.7 x10^{-3}	6
217979_at	*TSPAN13*	0.20	1.2 x10^{-7}	7
207821_s_at	*PTK2*	0.33	1.7 x10^{-4}	8
208820_at	*PTK2*	0.25	6.5 x10^{-4}	9
202481_at	*DHRS3*	0.60	3.7 x10^{-11}	10
201034_at	*ADD3*	0.30	1.4 x10^{-3}	11
201753_s_at	*ADD3*	0.31	8.9 x10^{-4}	12
201752_s_at	*ADD3*	0.36	3.2 x10^{-4}	13
205882_x_at	*ADD3*	0.34	7.4 x10^{-4}	14
203474_at	*IQGAP2*	-0.01	9.1 x10^{-1}	15
205884_at	*ITGA4*	0.45	8.3 x10^{-6}	16
205885_s_at	*ITGA4*	0.41	2.3 x10^{-5}	17
213416_at	*ITGA4*	0.53	2.6 x10^{-9}	18
211675_s_at	*MDFIC*	0.69	8.8 x10^{-12}	19
217599_s_at	*MDFIC*	0.53	7.6 x10^{-7}	20
210815_s_at	*CALCRL*	0.38	2.5 x10^{-3}	21
**204117_at**	***PREP***	**-0.23**	**2.7 x10^{-2}**	**22**
**37950_at**	***PREP***	**-0.29**	**8.2 x10^{-3}**	**23**
205273_s_at	*PITRM1*	0.04	7.1 x10^{-1}	24
219748_at	*TREML2*	0.25	1.6 x10^{-2}	25
204709_s_at	*KIF23*	-0.14	1.9 x10^{-1}	26
**213761_at**	***MDM1***	**-0.28**	**4.7 x10^{-3}**	**27**
**220397_at**	***MDM1***	**-0.33**	**4.2 x10^{-3}**	**28**
205352_at	*SERPINI1*	-0.12	2.4 x10^{-1}	29
200872_at	*S100A10*	0.48	1.2 x10^{-6}	30
**204731_at**	***TGFBR3***	**-0.22**	**2.7 x10^{-2}**	**31**
221282_x_at	*RUNX2*	0.06	5.5 x10^{-1}	32
**204671_s_at**	***ANKRD6***	**-0.38**	**3.7 x10^{-4}**	**33**
204672_s_at	*ANKRD6*	-0.08	4.5 x10^{-1}	34
**206301_at**	***TEC***	**-0.23**	**3.5 x10^{-2}**	**35**
206686_at	*PDK1*	-0.17	1.0 x10^{-1}	36
206887_at	*CCBP2*	0.14	2.4 x10^{-1}	37
204524_at	*PDPK1*	0.01	9.0 x10^{-1}	38
221244_s_at	*PDPK1*	-0.06	5.7 x10^{-1}	39
207735_at	*RNF125*	0.29	1.1 x10^{-2}	40
205038_at	*IKZF1*	0.14	3.0 x10^{-1}	41
205039_s_at	*IKZF1*	0.22	1.1 x10^{-1}	42
216901_s_at	*IKZF1*	0.18	1.5 x10^{-1}	43
213745_at	*ATRNL1*	0.26	4.9 x10^{-2}	44
218706_s_at	*GRAMD3*	0.48	4.1 x10^{-5}	45

Expression levels of IK target genes were compared in primary leukemic cells from 122 BCR-ABL$^+$ and 237 BCR-ABL$^-$ pediatric BPL patients (MILE Study, GSE13159). Transcript signal

values obtained from hybridization onto the Affymetrix Human Genome U133 Plus 2.0 Arrays were calculated using non-central trimmed mean of differences between perfect match and mismatch intensities with quantile normalization (DQN3, signal normalized with quartiles of the beta distribution with parameters p=1.2 and q=3. Of the 42 IK target transcripts representing 29 genes, only 7 transcripts representing 5 genes were significantly down regulated in BCR-ABL$^+$ patients. 25 transcripts representing 16 genes were expressed at significantly higher levels in leukemia cells from BCR-ABL$^+$ patients. Hierarchical cluster analysis identified *MDFIC* (0.69 SD units, P = 8.8 x 10^{-12}), *DHRS3* (0.6 SD units, P = 3.7 x 10^{-11}), *GSN* (0.49 SD units, P = 3.4 x 10^{-10}), *ITGA4* (0.53 SD units, P = 2.6 x 10^{-9}) and *TSPAN13* (0.2 SD units, P = 1.2 x 10^{-7}) as the most significantly up-regulated genes in the 122 BCR-ABL$^+$ patient samples.

Table S3. Primer Sets used for Amplifying and Sequencing *Ikaros/IKZF1* Exons 4, 5, 6 and 7 and their Exon-Intron Junctions

A. Genomic PCR Primers

Exon-Intron Junction	Sequence (5'->3')	Product Size (bp)	Upstream/Downstream from Exon (bp)
4	F: TCAAGGCTGAATGCACGGCG<br>R: TACCACTTGGAACCAATCGC	577	-169/+147
5	F: CCCAGCCAGTGAAGCGTTAA<br>R: CATACCAGCCTGACAAACGG	646	-175/+303
6	F: CAGGAATTTCACCAAGTCCG<br>R: CTAGGAGTTTCTCCCGATTG	533	-150/257
7	F: GTCATTGGCATTGCTCCGGC<br>R: CACTTCTCAGGGGTCAGTCA	785	-182/468

B.Sequencing PCR Primers

Sequencing Primer	Sequence (5'->3')	*IKZF1* Exon Number
IK-seqR4	TACCACTTGGAACCAATCGC	4
IK-seqF5	CCCAGCCAGTGAAGCGTTAA	5
IK-seqF6	CAGGAATTTCACCAAGTCCG	6
IK-seqR6	CTAGGAGTTTCTCCCGATTG	6
IK-seqF7N	GTCATTGGCATTGCTCCGGC	7

Table S4. Expression Levels of IK Target Genes in Primary Leukemia Cells from Matched-Pair Relapse vs. Diagnosis Specimens of Pediatric BPL Patients

Probeset ID	GeneSymbol	Fold Change (Relapse / Diagnosis, N=59)	Paired T-test P-value	Cluster Order
200696_s_at	*GSN*	0.96	0.731	1
203305_at	*F13A1*	0.94	0.631	2
200770_s_at	*LAMC1*	1.24	0.041	3
200771_at	*LAMC1*	1.18	0.093	4
219165_at	*PDLIM2*	1.11	0.201	5
204524_at	*PDPK1*	1.05	0.480	6
205038_at	*IKZF1*	1.10	0.324	7
205039_s_at	*IKZF1*	1.05	0.455	8
206301_at	*TEC*	1.08	0.259	9
203474_at	*IQGAP2*	1.00	0.967	10
213761_at	*MDM1*	1.05	0.662	11
204117_at	*PREP*	0.98	0.816	12
205273_s_at	*PITRM1*	1.06	0.579	13
37950_at	*PREP*	1.07	0.446	14
205885_s_at	*ITGA4*	1.13	0.299	15
210815_s_at	*CALCRL*	0.99	0.944	16
213416_at	*ITGA4*	0.91	0.544	17
217979_at	*TSPAN13*	1.00	0.991	18
201034_at	*ADD3*	1.11	0.460	19
201753_s_at	*ADD3*	0.96	0.767	20
201752_s_at	*ADD3*	1.14	0.307	21
205882_x_at	*ADD3*	1.09	0.458	22
220397_at	*MDM1*	1.02	0.889	23
207821_s_at	*PTK2*	1.22	0.064	24
219748_at	*TREML2*	0.97	0.817	25
202481_at	*DHRS3*	1.00	0.992	26
204731_at	*TGFBR3*	0.86	0.187	27
221282_x_at	*RUNX2*	1.02	0.791	28
206686_at	*PDK1*	1.17	0.168	29
207735_at	*RNF125*	1.05	0.705	30
208820_at	*PTK2*	1.06	0.664	31
206887_at	*CCBP2*	0.98	0.845	32
205884_at	*ITGA4*	1.04	0.807	33
214040_s_at	*GSN*	1.22	0.132	34
200872_at	*S100A10*	0.94	0.603	35
218706_s_at	*GRAMD3*	1.25	0.097	36
204671_s_at	*ANKRD6*	1.34	0.027	37
217599_s_at	*MDFIC*	1.12	0.465	38
211675_s_at	*MDFIC*	0.90	0.556	39
221244_s_at	*PDPK1*	0.88	0.317	40
216901_s_at	*IKZF1*	1.26	0.140	41
204672_s_at	*ANKRD6*	1.20	0.277	42
205352_at	*SERPINI1*	0.95	0.757	43
204709_s_at	*KIF23*	1.59	0.004	44
213745_at	*ATRNL1*	1.43	0.039	45

Matched pair gene expression values of IK target genes for leukemia cells obtained from 59 BPL patients at diagnosis and then at relapse (combined from GSE3912, N=32 and GSE18497, N=27). RMA-normalized values for the GSE18497 dataset and the MAS5- Signal intensity

values for the GSE3912 dataset were log_{10} transformed and mean-centered to the average value for the diagnosis samples for each gene transcript in each study. To determine the differential expression of each gene, paired T-tests were performed for the combined mean-centered values from GSE3910 and GSE18497 datasets (Unequal variance correction, P<0.05 deemed significant). We also compared the IK target gene expression levels in leukemic cells from initial diagnosis specimens of patients who subsequently experienced an early relapse (N=40; <36 months) versus a late relapse (N=19; >36months) (2-sample T-test).
